# What Is My Risk? A Mixed‐Methods Systematic Review of Risk Perception for Cardiometabolic Pregnancy Complications and Future Cardiometabolic Disease Development

**DOI:** 10.1111/obr.13967

**Published:** 2025-07-09

**Authors:** Elaine K. Osei‐Safo, Jennifer McIntosh, Shakira Onwuka, Sophia Torkel, Margaret McGowan, Kristie Cocotis, Caitlyn Angel, Sanjay Varatharaj, Helena Teede, Angela Melder, Sarah Lang, Lisa J. Moran

**Affiliations:** ^1^ Monash Centre for Health Research and Implementation, School of Clinical Sciences Monash University Clayton Victoria Australia; ^2^ Melbourne School of Population and Global Health University of Melbourne Carlton Victoria Australia; ^3^ Diabetes Victoria Carlton Victoria Australia; ^4^ Health and Social Care Unit Monash University Clayton Victoria Australia

**Keywords:** cardiovascular diseases, gestational diabetes, pregnancy‐induced hypertension, type 2 diabetes mellitus

## Abstract

**Introduction:**

Cardiometabolic pregnancy complications increase future cardiometabolic disease risk. Accurate risk perception plays a central role in adopting risk‐reducing lifestyle and health‐related behaviors, such as healthy eating, physical activity, and weight management. This review aimed to explore high‐risk pregnant and postpartum women's perception of their risk of developing cardiometabolic pregnancy complications or future cardiometabolic disease.

**Methods:**

Systematic search identified quantitative and qualitative data exploring risk perception in women (pregnant/postpartum) at risk of or diagnosed with gestational diabetes mellitus (GDM), hypertensive disorders of pregnancy (HDP), intrauterine growth restriction (IUGR), and preterm birth (PTB). A convergent integrated mixed‐methods synthesis was undertaken, with findings interpreted using the health belief and capability, opportunity, and motivation for behavior change models.

**Results:**

Overall, 84 studies were included, with the majority in GDM (77.4%) and HDP (23.8%), with limited research in PTB (8.3%) and IUGR (6.0%). Women had low–moderate knowledge of pregnancy complications as risk factors for future cardiometabolic disease and low–moderate perceived susceptibility to potential pregnancy complications and future cardiometabolic disease. Self‐perceived barriers, facilitators, cues to action, self‐efficacy, and self‐optimism impacted engagement with lifestyle and screening measures. The highest risk perception for future type 2 diabetes or cardiovascular disease was among women who had previously experienced GDM or HDP, respectively.

**Conclusion:**

Designing interventions to optimize women's risk perception will support informed decision‐making and empower women to make lifestyle changes to reduce future cardiometabolic risk.

## 1 Introduction

Cardiometabolic pregnancy complications are defined as maternal and fetal complications experienced during pregnancy whose pathophysiology is underpinned by various cardiometabolic‐related factors including impaired hemodynamic adaptation, endothelial and cardiac dysfunction, placental insufficiency, inflammation, and oxidative stress [1, 2]. They include gestational diabetes mellitus (GDM), hypertensive disorders of pregnancy (HDP) (e.g., preeclampsia [PE]), preterm birth (PTB), and intrauterine growth restriction (IUGR) and affect up to 30% of singleton pregnancies [2, 3]. Experiencing a cardiometabolic pregnancy complication presents a unique sex‐specific risk factor for future cardiometabolic disease development [4] which is associated with up to a 10‐fold increased risk of type 2 diabetes mellitus (T2DM) [5] and up to a twofold increased risk of cardiovascular disease (CVD) in the postpartum period and beyond [6, 7]. Evidence‐based guidelines recommend appropriate cardiometabolic screening, optimization of nutrition, physical activity, mental and emotional well‐being, and appropriate weight management to reduce the risk and severity of cardiometabolic pregnancy complications and future cardiometabolic disease (T2DM and CVD) [8, 9, 10, 11].

**FIGURE 1:**
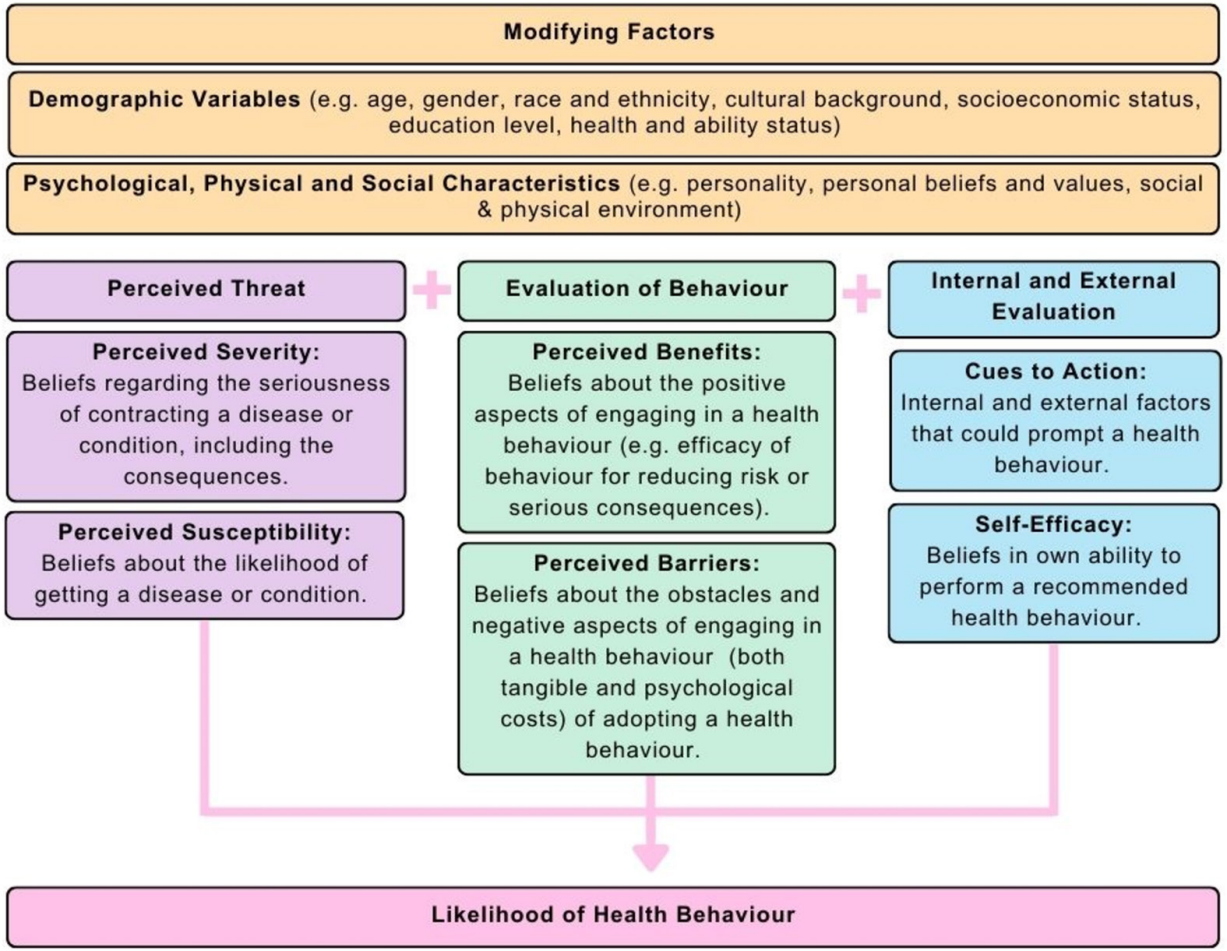
Health belief model (HBM). Modified from Etheridge et al. [16].

Risk perception refers to the judgment an individual makes about the severity and characteristics of a risk [12]. It is an individual belief about the potential harm or loss one may face and the likelihood of certain consequences associated with a particular behavior [12]. Three main factors influence perceived risk, including perceived likelihood (probability of harm), perceived susceptibility (vulnerability to harm), and perceived severity (the extent of possible harm) [12]. Risk perception is a core component of several health behavior theories and frameworks, including the health belief model (HBM) [13, 14, 15], in which risk perception is described as a key factor influencing an individual's engagement or disengagement in protective health behaviors [13, 15] (Figure 1). The capability, opportunity, and motivation model of behavior change (COM‐B) and associated theoretical domains framework (TDF) [17, 18] are also frequently used to understand factors influencing an individual's engagement or disengagement in protective health behaviors [17, 18]. It identifies three essential factors capable of changing behavior: capability, opportunity, and motivation [17, 18]. These models can be used to generate behavior change techniques as per the behavior change wheel (BCW), to inform intervention design [19, 20] or interventions to improve risk perception.

How women perceive their risks of cardiometabolic disease during pregnancy and after childbirth can influence their decisions to seek healthcare and engage in screening and preventive lifestyle measures before, during, and after pregnancy. Women at high risk of cardiometabolic pregnancy complications often have no perception of being at increased risk or perceive their risk for these complications as low [21]. Optimizing accurate risk perception is crucial to facilitate engagement with healthcare to change modifiable risk factors, including preventative lifestyle behaviors. Interventions that increase risk perception, awareness, and promote engagement with screening and healthy lifestyle behaviors will aid in the prevention of cardiometabolic pregnancy complications and future cardiometabolic disease. Understanding cardiometabolic risk perceptions among high‐risk women will inform the design of interventions to reduce their risk. As such, this systematic review aims to (a) explore pregnant women at risk or diagnosed with a cardiometabolic pregnancy complication's (GDM, HDP, IUGR, and PTB) perceived risk of developing or managing cardiometabolic pregnancy complications and (b) explore postpartum women previously diagnosed with a cardiometabolic pregnancy complication's (GDM, HDP, IUGR, and PTB) perceived risk of developing or managing future cardiometabolic disease (CVD and T2DM).

## 2 Methods

A mixed‐methods systematic review of quantitative and qualitative data from cross‐sectional studies, qualitative studies, and mixed‐methods studies was performed. The protocol was registered in PROSPERO (registration number: CRD42022343277) in June 2022. The review was performed according to the Preferred Reporting Items for Systematic Reviews and Meta‐Analysis (PRISMA) protocol [22].

### 2.1 Search Methods

A systematic search was conducted up to June 13, 2023, using manual citation searching. Ovid MEDLINE, CINAHL Plus, Cochrane Library, and APA PsychInfo were searched. The search strategy (Data S1) was developed using key words and subject headings related to risk perception in pregnant/postpartum women, risk communication among health professionals, and cardiometabolic pregnancy complications (GDM, HDP, IUGR/FGR, and SPTB/PTB). A separate systematic review linked to the PROSPERO will report the risk communication results.

### 2.2 Selection Process

Search results were managed using Covidence. Following the removal of duplicate studies, four reviewers (100% E.O., 45% S.L., 45% S.T., and 10% S.V.) screened the title, abstract, and keywords of all studies in duplicate. Full‐text screening was conducted in duplicate by five reviewers (100% E.O., 46% M.M., 23% K.C., 23% F.T., and 8% S.V.). Discrepancies between reviewers were resolved by consensus or discussed with an additional review author.

Quantitative, qualitative, and mixed‐methods research was included. Participants were pregnant women at risk of (as defined by the Australian Clinical Practice Guidelines for Pregnancy Care [23]) one or more cardiometabolic pregnancy complication (GDM, HDP, IUGR, and PTB) or pregnant/postpartum women medically diagnosed with one or more cardiometabolic pregnancy complication (GDM, HDP, IUGR, and PTB). Studies exploring pregnant women's perceived risk of developing or managing cardiometabolic pregnancy complications were included. Studies exploring postpartum women with prior cardiometabolic pregnancy complication's perceived risk of developing or managing future cardiometabolic disease (CVD and T2DM) were also included. “Postpartum” was defined as relating to or denoting the period after childbirth, with no limit on the time period. “Future cardiometabolic disease risk” included both short (less than 5 years) and long‐term T2DM and/or CVD risk (more than 5 years).

### 2.3 Data Extraction

Data extraction was completed by one review author (E.O.). This included author, title, year of publication, study design, study aims, participant characteristics (participant number, participant type [pregnant/postpartum women, age, race/ethnicity, socioeconomic status [SES], parity, education level, and cardiometabolic pregnancy complication(s)]), how risk perception was assessed, and study findings (quantitative and qualitative risk perception data).

### 2.4 Quality Appraisal

Quality appraisal was conducted with 25% cross‐checking (85% C.A., 15% S.V., and 25% E.O.). Discrepancies between reviewers were resolved by consensus or discussed with an additional review author. The following quality appraisal tools were used: Critical Appraisal Skills Program (CASP) Qualitative Studies Checklist [24], Center for Evidence‐Based Management (CEMB) Critical Appraisal Checklist for a Cross‐Sectional Study [25], and the Mixed Methods Appraisal Tool (MMAT) [26].

### 2.5 Data Analysis and Synthesis

A convergent integrated approach for the analysis of mixed‐methods systematic reviews [27] was utilized. Initially, data were transformed into a mutually compatible format via converting quantitative data into narrative descriptions. For example, a results table that displayed the percentage of women who ticked “low risk” on a survey as 80% was narratively described as “80% of women perceive their risk as low” and qualitatively coded, regardless of significance. All data were inductively coded and organized into categories. Categories were then mapped to an a priori coding framework combining the HBM [13, 14], COM‐B, and TDF [17, 18] (Figure S1). The HBM constructs within the framework included perceived severity, perceived susceptibility, perceived benefits, perceived barriers, cues to action, and self‐efficacy. The COM‐B constructs and their associated TDF domains within in the framework included capability (physical and psychological), opportunity (social and physical), and motivation (automatic and reflective) [17, 18]. These frameworks were used to concurrently appraise data as they collectively explore one's perceived personal risk and susceptibility to disease and illness (HBM) and factors that may influence behavior change (COM‐B). Similar categories were aggregated to produce the overall integrated findings [27]. All coding was managed using QSR International NVivo 14 software 2023.

Key research findings for each construct of the HBM (and associated COM‐B constructs) were synthesized from the overall results and then mapped to intervention functions and behavior change techniques (BCTs) as per the BCW [19, 20]. This was done to methodically generate suggested practice recommendations for interventions to optimize the risk perception and management of cardiometabolic disease during and after pregnancy in high‐risk women.

## 3 Results

**FIGURE 2:**
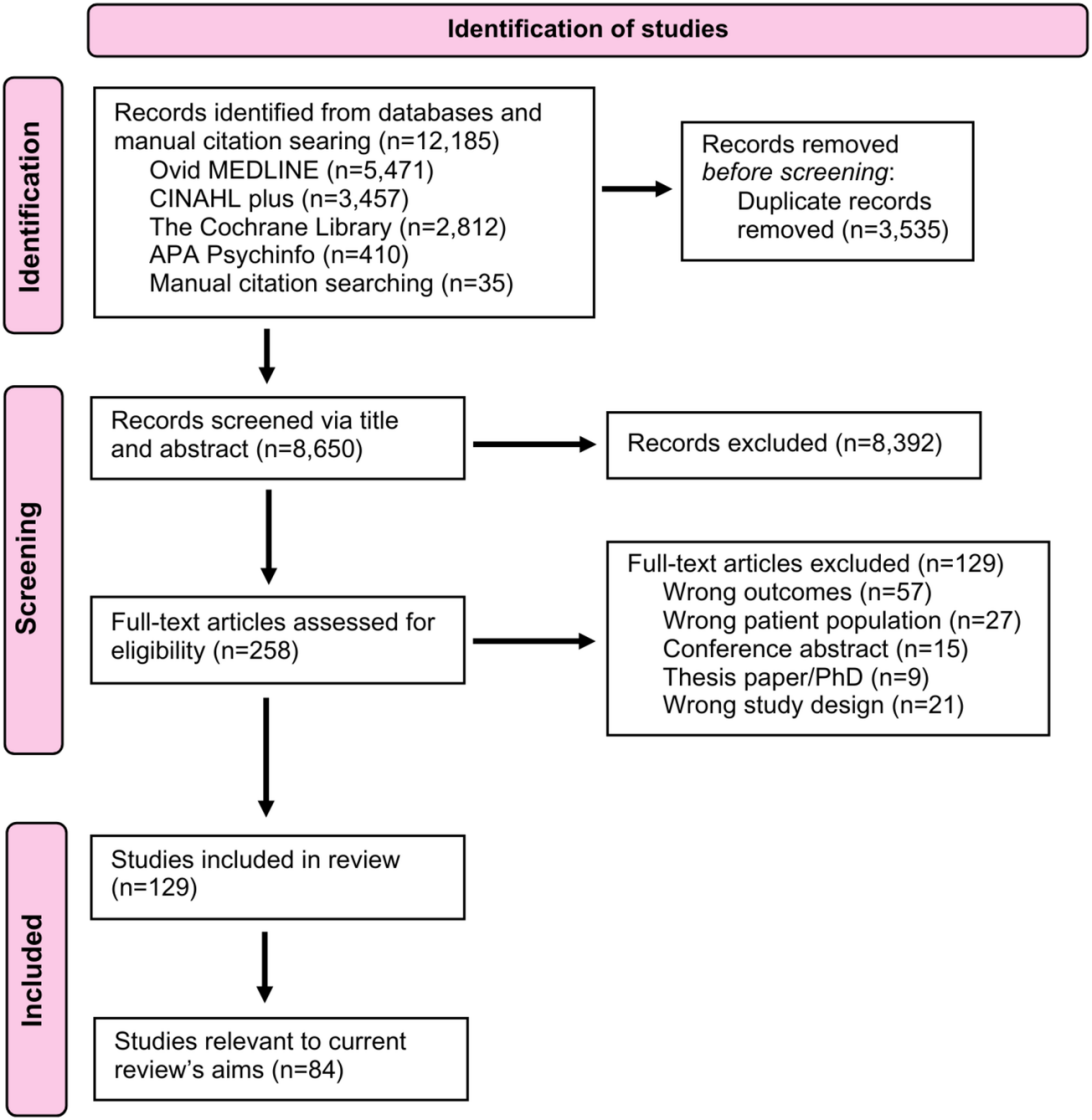
PRISMA flow diagram.

The search identified 12,185 records. After full‐text screening, 129 studies met the inclusion criteria, 84 of which pertain to the current review's aims (Figure 2).

### 3.1 Study Characteristics

A total of 10,548 women's perspectives were obtained across the 84 included studies from 25 countries (Table 1). Study designs were qualitative (*n* = 39) [28, 29, 33, 35, 37, 38, 42, 43, 44, 46, 49, 50, 52, 55, 57, 58, 59, 60, 61, 62, 72, 73, 79, 81, 83, 87, 89, 90, 92, 93, 95, 96, 97, 99, 102, 103, 106, 107, 108], quantitative cross‐sectional (*n* = 38) [30, 31, 32, 34, 36, 40, 41, 45, 47, 48, 51, 53, 54, 56, 63, 64, 68, 69, 70, 71, 74, 75, 76, 77, 78, 82, 84, 85, 86, 88, 91, 94, 100, 104, 105, 109, 110, 111], and mixed methods (*n* = 7) [39, 65, 66, 67, 80, 98, 101]. The most common cardiometabolic pregnancy complication reported on was GDM (*n* = 65 studies [77.4%], *n* = 7992 participants), followed by HDP (*n* = 20 studies [23.8%], *n* = 2443 participants), PTB (*n* = 7 studies [8.3%], *n* = 1127 participants), and IUGR (*n* = 5 studies [6.0%], *n* = 561 participants). The most commonly reported on cardiometabolic disease was T2DM (*n* = 59 studies [70.2%], *n* = 7378 participants) followed by CVD (*n* = 18 studies [21.4%], *n* = 1900 participants). The majority of studies were conducted in the United States (*n* = 25), Australia (*n* = 18), and the United Kingdom (*n* = 7). The quality assessment found study quality was high (*n* = 43), moderate (*n* = 20), and low (*n* = 21) quality (Tables S2–S4). Quality was primarily downgraded due to a lack of acknowledgment of the relationship between researcher(s) and participant(s) (qualitative studies), recruitment and data collection methods bias (quantitative), possible confounding factors that were not accounted for (quantitative), sample size not based on prestudy consideration of statistical power (quantitative), and participants not representing the population the findings are referred to (quantitative).

**TABLE 1:** Characteristics of included studies (*n* = 84).

Author, year	Country of origin	Cardiometabolic pregnancy complication(s) of interest (GDM, HDP, IUGR, and PTB)	Postpartum cardiometabolic disease(s) of interest (T2DM and CVD)	Study design	Relevant study sample	How was risk perception assessed?	Quality assessment (low/moderate/high)
Abraham and Wilk, 2014 [28]	USA	GDM	T2DM	Qualitative	10 postpartum women with previous GDM	Semistructured interview questions	High
Adu‐Bonsaffoh et al., 2023 [29]	Ghana	HDP	CVD	Qualitative	22 pregnant women with HDP	Semistructured interview questions and focus group questions	High
Akinwaare et al., 2020 [30]	Nigeria	GDM	NA	Quantitative	182 pregnant women at risk of GDM	Survey, 4‐point scale (1 = *least risk*, 4 = *high risk*)	Moderate
Aldridge et al., 2023 [31]	Australia	GDM, HDP, IUGR, and PTB	CVD	Quantitative	188 postpartum women with previous GDM, HDP, IUGR, and PTB	Survey, 2 answers (“yes” and “no”)	Low
Atkinson et al., 2023 [32]	Australia	HDP	CVD	Quantitative	438 postpartum women with previous HDP	Survey, 2 answers (“Yes, I am aware of this” and “No, I did not know”)	Low
Bagger et al., 2021 [33]	Denmark	GDM	T2DM	Qualitative	12 postpartum women with prior GDM	Semistructured interview questions	High
Bayrampour et al., 2012 [34]	Canada	PTB and IUGR	NA	Quantitative	54 pregnant women at risk of PTB and IUGR	Survey, nine‐item scale with visual analog scales from 0 to 100. Higher scores indicate higher levels of perceived risk.	Moderate
Bennett et al., 2011 [35]	Germany	GDM	T2DM	Qualitative	22 postpartum women with previous GDM	Semistructured interview questions	High
Beussink‐Nelson et al., 2022 [36]	USA	GDM, HDP, IUGR, and PTB	CVD	Quantitative	182 postpartum women with previous GDM, HDP, IUGR, and PTB	Survey, 5‐point scale (*much lower than average*, *somewhat lower than average*, *average*, *somewhat higher than average*, and *much higher than average*)	Low
Bogale et al., 2021 [37]	Norway	HDP, GDM, and IUGR	NA	Qualitative	18 pregnant women with or at risk of HDP, GDM, and IUGR	Semistructured interview questions	Low
Brown et al., 2013 [38]	UK	HDP	CVD	Qualitative	12 postpartum women with previous HDP	Semistructured interview questions	High
Brown et al., 2017 [39]	USA	GDM	T2DM	Mixed methods	35 postpartum women with previous GDM	Focus group questions and a survey (4‐point scale, 1 = *almost no chance*, 4 = *high chance*)	High
Burgess and Feliu, 2019 [40]	USA	HDP	CVD	Quantitative	241 postpartum women with previous HDP	Survey, 2 answers (*aware* and *unaware*)	Low
Chuang et al., 2008 [41]	USA	PTB	NA	Quantitative	417 pregnant women at risk of PTB	Survey, 4‐point scale (*very likely*, *somewhat likely*, *somewhat unlikely*, and *very unlikely*)	Moderate
Collier et al., 2011 [42]	USA	GDM	T2DM	Qualitative	89 pregnant and postpartum women with GDM	Focus group questions	Moderate
Dennison et al., 2022 [43]	UK	GDM	T2DM	Qualitative	20 postpartum women with previous GDM	Semistructured interview questions	High
Dijkhuis et al., 2020 [44]	Netherlands	HDP	CVD	Qualitative	15 postpartum women with previous HDP	Semistructured interview questions	High
Dobbs et al., 2021 [45]	USA	PTB	NA	Quantitative	6 pregnant women at risk of PTB	Survey, 10‐point scale (1 = *not at all likely*, 10 = *very likely*)	Low
Eades et al., 2020 [46]	UK	GDM	T2DM	Qualitative	282 pregnant women with GDM	Online discussion board topics	High
Feig et al., 1998 [47]	Canada	GDM	T2DM	Quantitative	65 postpartum women with previous GDM	Survey, 4‐point scale (*not at all possible*, *somewhat possible*, *very possible*, highly *possible*)	Low
Ferranti et al., 2014 [48]	USA	GDM	T2DM	Quantitative	75 postpartum women with previous GDM	Survey, 4‐point scale (*almost no chance*, *slight chance*, *moderate chance*, *high chance*)	Low
Ge et al., 2016 [49]	China	GDM	T2DM	Qualitative	15 pregnant women with GDM	Semistructured interview questions	High
Ge et al., 2016 [50]	China	GDM	T2DM	Qualitative	17 pregnant women with GDM	Semistructured interview questions	High
Goldstein et al., 2015 [51]	Australia	GDM	T2DM	Quantitative	46 pregnant women with GDM	Survey, multiple options provided (did not specify options)	Low
Graco et al., 2009 [52]	Australia	GDM	T2DM	Qualitative	10 postpartum women with previous GDM	Semistructured interview questions	High
Gray et al., 2020 [53]	Australia	GDM	T2DM	Quantitative	429 postpartum women with previous GDM	Survey, 4‐point scale (*no risk*, *low risk*, *moderate risk*, *high risk*)	Moderate
Gray et al., 2021 [54]	Australia	GDM	T2DM	Quantitative	121 postpartum women with previous GDM	Survey, 4‐point scale (*no, I am not at risk*, *yes, low risk*, *yes, moderate risk*, *yes, high risk*)	Low
Gunn et al., 2020 [55]	USA	GDM	T2DM	Qualitative	29 postpartum women with previous GDM	Semistructured interview questions	High
Harrison et al., 2012 [56]	Australia	GDM	NA	Quantitative	97 pregnant women at risk of GDM	Survey, 4‐point scale (*no risk*, *slight risk*, *moderate risk*, *high risk*)	High
Hirst et al., 2012 [57]	Vietnam	GDM	T2DM	Qualitative	35 pregnant women with GDM	Focus group questions	High
Hjelm et al., 2005 [58]	Sweden	GDM	T2DM	Qualitative	27 pregnant women with GDM	Semistructured interview questions	High
Hjelm et al., 2009 [59]	Sweden	GDM	T2DM	Qualitative	27 postpartum women with previous GDM	Semistructured interview questions	High
Hjelm et al., 2012 [60]	Sweden	GDM	T2DM	Qualitative	23 pregnant and postpartum women with GDM	Semistructured interview questions	High
Hjelm et al., 2021 [61]	Sweden	GDM	T2DM	Qualitative	9 pregnant and postpartum women with GDM	Semistructured interview questions	Moderate
Hjelm et al., 2022 [62]	Sweden	GDM	T2DM	Qualitative	9 pregnant and postpartum women with GDM	Semistructured interview questions	High
Huang et al., 2022 [63]	USA	GDM	T2DM	Quantitative	106 postpartum women with previous GDM	Survey, 4‐point scale (*almost no risk*, *slight risk*, *moderate risk*, *high risk*)	Moderate
Hutchesson et al., 2018 [64]	Australia	HDP	CVD	Quantitative	127 postpartum women with previous HDP	Survey, 3 answers (“true,” “false,” and “unsure”)	Low
Jelsma et al., 2016 [65]	Netherlands, Belgium, UK, Austria, Denmark, Ireland, Italy, Poland, and Spain	GDM	T2DM	Mixed methods	92 pregnant and postpartum women with GDM	Semistructured interview questions and a survey (5‐point scale, *strongly agree*, *agree*, *neutral*, *disagree*, *strongly disagree*)	High
Jones et al., 2012 [66]	USA	GDM	T2DM, CVD	Mixed methods	22 postpartum women with previous GDM	Semistructured interview questions and a survey (4‐point scale, *almost no risk*, *slight risk*, *moderate risk*, *high risk*)	High
Jones et al., 2015 [67]	USA	GDM	T2DM, CVD	Mixed methods	26 postpartum women with previous GDM	Semistructured interview questions, focus group questions and a survey (4‐point scale, *almost no chance*, *slight chance*, *moderate chance*, *high chance*)	High
Joshi et al., 2020 [68]	Ghana	HDP	CVD	Quantitative	150 postpartum women with previous HDP	Survey, 3 answers (“yes,” “no,” and “I do not know”)	Moderate
Kaiser et al., 2016 [69]	Switzerland	GDM	T2DM	Quantitative	173 pregnant and postpartum women with GDM	Survey, 4‐point scale (1 = *almost no risk*, 4 = *maximum risk*)	Moderate
Kim et al., 2007 [70]	USA	GDM	T2DM	Quantitative	217 postpartum women with previous GDM	Survey, 4‐point scale (*almost no chance*, *a slight chance*, *a moderate chance*, *a high chance*)	Low
Kim et al., 2020 [71]	Bangladesh	GDM	T2DM	Quantitative	368 pregnant women with or at risk of GDM	Survey, 5‐point scale (*strongly agree* to *strongly disagree*)	Moderate
Krompa et al., 2020 [72]	France	GDM	T2DM	Qualitative	16 postpartum women with previous GDM	Semistructured interview questions	High
Lucas et al., 2022 [73]	USA	GDM	T2DM	Qualitative	36 postpartum women with previous GDM	Semistructured interview questions	Moderate
Malcolm et al., 2009 [74]	Canada	GDM	T2DM	Quantitative	77 postpartum women with previous GDM	Survey, 3 answers (“same as other women or unsure of future risk,” “increased a little,” and “increased a lot”)	Low
Mekie et al., 2021 [75]	Ethiopia	HDP	NA	Quantitative	423 pregnant women at risk of HDP	Survey, 3 answers (“agree,” “disagree,” and “unsure”)	Moderate
Minsart et al., 2014 [76]	Belgium	GDM	T2DM	Quantitative	72 postpartum women with previous GDM	Survey, 4‐point scale (*no risk*, *low risk*, *moderate risk*, *high risk*)	Moderate
Morrison et al., 2010 [77]	Australia	GDM	T2DM	Quantitative	1176 postpartum women with previous GDM	Survey, 5‐point scale (*very low risk*, *low risk*, *moderate risk*, *high risk*, *very high risk*)	Low
Mukerji et al., 2016 [78]	Canada	GDM	T2DM	Quantitative	614 pregnant women with GDM	Survey, 4‐point scale (*almost no chance*, *slight chance*, *moderate chance*, *high chance*)	Moderate
Nedergaard et al., 2023 [79]	Denmark	GDM	T2DM	Qualitative	20 postpartum women with previous GDM	Semistructured interview questions	High
Nicklas et al., 2011 [80]	USA	GDM	T2DM	Mixed methods	38 postpartum women with previous GDM	Semistructured interview questions and focus group questions	High
Nielsen et al., 2022 [81]	Denmark	HDP	CVD	Qualitative	10 postpartum women with previous HDP	Semistructured interview questions	High
Nitert et al., 2011 [82]	Australia	GDM and HDP	NA	Quantitative	115 pregnant women at risk of GDM and HDP	Survey, 5‐point scale (*very low risk*, *low risk*, *average risk*, *high risk*, *very high risk*, in addition to a “do not know” option)	Moderate
Offomiyor and Rehal, 2023 [83]	Nigeria	GDM	T2DM	Qualitative	22 pregnant women at risk of GDM	Semistructured interview questions	Moderate
Okely et al., 2019 [84]	UK	GDM	T2DM	Quantitative	59 pregnant women with GDM	Survey, 5‐point scale (*no risk at all*, *quite low risk*, *medium risk*, *quite high risk*, *very high risk*)	Low
O'Reilly et al., 2021 [85]	Australia	GDM	T2DM	Quantitative	745 postpartum women with previous GDM	Survey, 2‐point scale (*low risk*, *high risk*)	Moderate
Park et al., 2018 [86]	Korea	GDM	T2DM	Quantitative	237 postpartum women with previous GDM	Survey, 3 answers (“yes,” “no,” and “do not know”)	Moderate
Parsons et al., 2019 [87]	UK	GDM	T2DM	Qualitative	50 postpartum women with previous GDM	Semistructured interview questions and focus group questions	High
Passey et al., 2012 [88]	Australia	IUGR	NA	Quantitative	119 pregnant women at risk of IUGR	Survey, 3 answers (“agree,” “disagree,” and “not sure”)	High
Poth and Carolan, 2013 [89]	Australia	GDM	NA	Qualitative	6 pregnant women at risk of GDM	Semistructured interview questions	High
Qian et al., 2022 [90]	China	GDM	T2DM	Qualitative	16 postpartum with previous GDM	Semistructured interview questions	High
Qian et al., 2023 [91]	China	GDM	T2DM	Quantitative	324 postpartum women with previous GDM	Survey, 3 answers (“true,” “false,” and “I do not know”)	Low
Razee et al., 2010 [92]	Australia	GDM	T2DM	Qualitative	57 postpartum women with previous GDM	Semistructured interview questions	High
Rossiter et al., 2022 [93]	Australia	HDP	CVD	Qualitative	34 postpartum with previous HDP	Semistructured interview questions	High
Roth et al., 2020 [94]	Australia	HDP	T2DM and CVD	Quantitative	174 postpartum women with previous HDP	Survey, 3 answers (“greater than,” “less than,” or “equal to” that of a woman with a normotensive pregnancy)	Low
Seely et al., 2013 [95]	USA	HDP	CVD	Qualitative	20 postpartum women with previous HDP	Focus group questions	High
Shang et al., 2021 [96]	China	GDM	T2DM	Qualitative	20 pregnant and postpartum with GDM	Semistructured interview questions	High
Sharma et al., 2019 [97]	UK	GDM	T2DM	Qualitative	20 postpartum women with previous GDM	Semistructured interview questions	High
Singh et al., 2018 [98]	USA	GDM	T2DM	Mixed methods	564 pregnant women with GDM	Semistructured interview questions	High
Skurnik et al., 2016 [99]	USA	HDP	CVD	Qualitative	14 postpartum women with previous HDP	Focus group questions	High
Stacy et al., 1994 [100]	USA	PTB	NA	Quantitative	134 pregnant women at risk of PTB	Survey, 3 choices (“agree,” “disagree,” and “not sure”)	Low
Sterne et al., 2011 [101]	Australia	GDM	T2DM	Mixed methods	88 postpartum women with previous GDM	Survey with open‐ended questions	High
Stotz et al., 2019 [102]	USA	GDM	T2DM	Qualitative	5 postpartum women with previous GDM	Semistructured interview questions	High
Sunny et al., 2020 [103]	Singapore	GDM	T2DM	Qualitative	20 postpartum women with previous GDM	Semistructured interview questions	High
Sutherland et al., 2020 [104]	USA	HDP and GDM	T2DM and CVD	Quantitative	79 postpartum women with previous GDM, HDP	Survey, 5‐point scale (1 = *completely disagree*, 5 = *completely agree*)	Low
Swan et al., 2007 [105]	Australia	GDM	T2DM	Quantitative	53 postpartum women with previous GDM	Survey, 6‐point scale (*strongly agree* to *strongly disagree*)	Moderate
Tang et al., 2015 [106]	USA	GDM	T2DM	Qualitative	23 postpartum women with previous GDM	Semistructured interview questions	High
Teh et al., 2021 [107]	Singapore	GDM	T2DM	Qualitative	14 postpartum women with previous GDM	Semistructured interview questions	High
Toft et al., 2022 [108]	Norway	GDM	T2DM	Qualitative	14 postpartum women with previous GDM	Semistructured interview questions	High
Traylor et al., 2016 [109]	USA	HDP, PTB	CVD	Quantitative	146 pregnant and postpartum women with HDP, PTB	Survey, 4‐point scale (*almost no risk*, *slight risk*, *moderate risk*, *high risk*), 2 answer responses (“yes” and “no”), 4 answer responses (“increases risk,” “has no effect on risk,” “decreases risk,” “do not know”)	Low
Vu et al., 2022 [110]	USA	GDM	T2DM	Quantitative	264 postpartum women with previous GDM	Survey, 4‐point scale (“almost no chance,” “slight chance,” “moderate chance,” “high chance”)	Moderate
Zera et al., 2013 [111]	USA	GDM	T2DM	Quantitative	70 postpartum women with GDM	Survey, 4‐point scale (*almost no chance*, *slight chance*, *moderate chance*, *high chance*)	Low

Abbreviations: CVD: cardiovascular disease, GDM: gestational diabetes mellitus, HDP: hypertensive disorders of pregnancy, IUGR: intrauterine growth restriction, NA: not applicable, PTB: preterm birth, T2DM: type 2 diabetes mellitus, UK: United Kingdom, USA: United States of America.

### 3.2 Integrated Findings

High‐risk pregnant and postpartum women's perceived risk of developing or managing cardiometabolic pregnancy complications or cardiometabolic disease after birth was synthesized and mapped onto the six constructs of the HBM (perceived susceptibility, perceived severity, perceived benefits, perceived barriers, cues to action, and self‐efficacy) [13, 14] with integration of the COM‐B (capability, opportunity, and motivation) [17, 18].

#### 3.2.1 High‐Risk Pregnant Women

Studies explored risk perceptions of pregnant women: at risk of GDM (*n* = 10), HDP (*n* = 4), IUGR (*n* = 3), and PTB (*n* = 5) and diagnosed with GDM (*n* = 15), HDP (*n* = 3), IUGR (*n* = 1), and SPTB (*n* = 1).

##### 3.2.1.1 Perceived Susceptibility

Perceived susceptibility refers to one's beliefs about their likelihood of getting a disease or condition [13, 14].

###### 3.2.1.1.1 Perceived Susceptibility for Developing GDM, HDP, IUGR, and PTB in At‐Risk Women

Among women at risk of developing a cardiometabolic pregnancy complication, the perceived susceptibility of developing GDM varied across studies [30, 33, 37, 51, 56, 65, 82, 108]. Some studies reported that up to 50% of high‐risk women did not perceive themselves as susceptible to developing GDM [56, 108], while others showed that 33%–87.8% of women perceived themselves as high risk [30, 51, 56, 65, 82]. Women at risk of HDP had both low [29, 37] and high [82, 88, 94, 109] perceived susceptibility. Where quantified, 88.2%–90.0% of women perceived their risk of HDP as high [82, 94]. Perceived susceptibility of developing IUGR was also a variable, reported as low [37], moderate [45], and high [88, 100], with 79.6% of women perceiving themselves as high risk in one study [100]. Perceived susceptibility for PTB was reported as moderate in one study [45] or ranged from 22.2% to 62.8% of women perceiving themselves as high risk [34, 41, 82].

###### 3.2.1.1.2 Knowledge of GDM, HDP, IUGR, and PTB in At‐Risk or Diagnosed Women

Women at risk of or diagnosed with a cardiometabolic pregnancy complication reported varied understanding of each condition, with knowledge influencing their perceived susceptibility to developing a cardiometabolic pregnancy complication. Women often lacked knowledge of GDM, including definition, pathogenesis, symptoms, and management [30, 50, 71, 83, 89, 90, 102, 106, 108]. However, one study suggested most women had good overall knowledge of GDM [86]. Risk factors identified by women included having a higher weight [33, 46, 51, 82, 83, 89, 92, 108], suboptimal diet [33, 37, 46, 49, 50, 57, 83, 89, 90, 92, 108], sedentary behavior [33, 37, 83, 92], stress [50], high blood glucose levels [89], family history, and genetic susceptibility [33, 50, 57, 83, 89, 108]. Some women attributed GDM largely to biological factors such as hormones and genetics [46, 92] or were uncertain about risk factors for GDM [57, 60, 89, 102, 108], which was attributed by some women to inadequate risk communication by healthcare professionals [108].

Overall knowledge of HDP ranged from poor (22.4% of women), moderate (48.8%), and good (28.8%) in one study [75]. Some women did not understand HDP well enough to explain it to someone else [29, 68] with limited understanding contributing to fears of potential consequences [81]. Some women had good knowledge of HDP risk factors, including suboptimal diet [37, 38], sedentary behavior [37, 38], having a higher weight [38, 82], smoking [38, 88], stress [29], family history [38], and previous or current obstetric complications (e.g., HDP and GDM) [83, 86, 94]. Women identified advanced maternal age [34], having a higher weight [82], and smoking [88, 100] as risk factors for PTB. In the limited research, overall knowledge of IUGR was mixed [37]. Overall knowledge of GDM, HDP, and IUGR was reported as dependent on education, parity, medical history, and knowing someone who had experienced a pregnancy complication [37].

###### 3.2.1.1.3 Link Between Knowledge and Risk Perception in At‐Risk or Diagnosed Women

Greater knowledge of cardiometabolic pregnancy complications in women at risk of GDM, PTB, and IUGR was associated with higher risk perception, whereas less knowledge was associated with lower risk perception [30, 34]. In women with prior GDM [86], HDP [75, 94] and PTB [41] previous obstetric complications were associated with greater knowledge about and higher perceived risk for developing a cardiometabolic pregnancy complication in subsequent pregnancies.

###### 3.2.1.1.4 Knowledge of Screening in At‐Risk or Diagnosed Women

Knowledge of screening during pregnancy was low among women at risk of or diagnosed with GDM, HDP, and IUGR, although there was limited research [37, 57]. Women reported poor understanding about reasons for testing for GDM [57] and low awareness and perception of the importance of antenatal screening for GDM, HDP, and IUGR [37].

###### 3.2.1.1.5 Knowledge of Preventative Lifestyle Behaviors in At‐Risk or Diagnosed Women

Women at risk or diagnosed with a cardiometabolic pregnancy complication suggested behaviors that could reduce their risk of developing GDM, HDP, and IUGR, including a positive mental attitude [49, 62], healthy diet [37, 38, 51, 62, 86, 89, 90, 92], regular physical activity [37, 38, 51, 62, 86, 89, 90, 92], weight management [38, 82, 92], abstaining from alcohol and smoking [38], managing blood glucose levels [38, 90], stress management [89], adequate sleep [89], a healthy work–life balance [89], pregnancy planning [37, 38, 75], early healthcare seeking [37, 38, 75], and regular check‐ups [37, 38, 75]. While some studies reported the majority of women had poor knowledge and lacked awareness of preventative measures for GDM and HDP [30, 68, 83, 102], other studies suggested women have some knowledge but lack understanding of the physiological mechanisms that reduce the risks of developing GDM [73, 102].

##### 3.2.1.2 Perceived Severity

Perceived severity refers to one's beliefs regarding the seriousness and consequences of contracting a disease or condition [13, 14].

###### 3.2.1.2.1 Perceived Severity of Cardiometabolic Pregnancy Complications in At‐Risk or Diagnosed Women

Some women at risk of or diagnosed with GDM (*n* = 19) [33, 37, 42, 49, 50, 51, 57, 58, 60, 71, 73, 83, 84, 86, 89, 90, 91, 92, 106], HDP (*n* = 5) [37, 38, 68, 81, 94], and IUGR (*n* = 1) [37] reported beliefs about negative health consequences relating to themselves and their fetus/baby. Women variably perceived the risks to themselves as not severe [49, 50, 60, 81, 90], were unclear of the severity [37, 42, 57], or perceived cardiometabolic pregnancy complications as severe illnesses that may result in additional pregnancy complications or future cardiometabolic complications in subsequent pregnancies and postpartum [33, 38, 42, 51, 58, 60, 68, 71, 83, 84, 86, 89, 91, 94, 106]. Beliefs about the consequences of GDM for one's fetus/baby included premature birth [57, 73, 89, 90], still birth and fetal death [42, 57, 92], macrosomia [42, 50, 51, 57, 73, 83, 86, 89, 90, 91], neonatal hypoglycemia [50, 51, 57, 71, 73, 86, 90], birth defects, developmental disruptions, and abnormalities [42, 50, 57, 73, 89, 90, 92].

###### 3.2.1.2.2 Emotional Impact of Being High‐Risk, Developing, and Managing Cardiometabolic Pregnancy Complications

Several studies described the myriad of mental and emotional health impacts of a cardiometabolic pregnancy complication diagnosis among women at risk of or diagnosed with GDM (*n* = 18) [28, 33, 46, 49, 50, 57, 58, 60, 62, 65, 72, 84, 87, 90, 92, 96, 98, 102, 108] or HDP (*n* = 2) [29, 81]. Women often felt abandoned by the healthcare system following diagnosis and reported receiving insufficient support to understand their test results, health implications, and management [28, 29]. Women described feeling overwhelmed [28, 46], anxious [28, 57, 81, 84, 90], panicked [58, 72], shocked and surprised [28, 33, 60, 72, 81, 98, 108], sad and disappointed [28, 33, 46, 49, 58, 60, 84, 98], stressed [72, 90, 92], and afraid, worried, and concerned [28, 33, 46, 50, 57, 58, 60, 62, 72, 81, 90, 92, 102]. These emotions related to their diagnosis, implications for their own and fetus/baby's health, and management including balancing their own dietary and fetal nutritional needs. Women also felt stigmatized [28, 50, 108], guilty, ashamed, embarrassed, blamed themselves for their diagnosis, and feared being judged by healthcare professionals, social connections, and family in response to diagnosis [28, 46, 57, 87, 96, 102, 108]. A family history of cardiometabolic disease left some women feeling powerless in their ability to change their risk [102]. Despite this, some pregnant women diagnosed with GDM felt motivated and empowered to engage in healthful behaviors to better manage their condition and minimize risks for themselves and their fetus/baby following diagnosis [46, 65, 96].

##### 3.2.1.3 Perceived Benefits

Perceived benefits refer to one's beliefs about positive aspects of engaging in a health behavior [13, 14]. Women at risk or diagnosed with GDM (*n* = 8) [37, 42, 49, 51, 62, 82, 89, 92], HDP (*n* = 3) [37, 75, 82], and IUGR (*n* = 1) [37] often perceived that healthy behaviors during pregnancy would significantly improve pregnancy outcomes, decrease the risk of cardiometabolic pregnancy complications, improve complication management, and reduce the risk of adverse birth and infant outcomes (e.g., macrosomia, cesarean section delivery, and still birth) [37, 42, 49, 51, 62, 75, 82, 89, 92].

##### 3.2.1.4 Perceived Barriers

Perceived barriers refer to one's beliefs about the obstacles and negative aspects of engaging in a health behavior [13, 14]. The level of adherence to a healthy lifestyle varied among women at risk of or diagnosed with GDM, HDP, and IUGR [37, 83, 84, 90]. Barriers to engaging in preventative action and healthy lifestyle management according to core constructs within the COM‐B model are summarized in Table 2.

**TABLE 2:** Barriers to adopting a healthy lifestyle for the prevention and management of cardiometabolic pregnancy complications and future cardiometabolic disease.

Relevant COM‐B domain	Perceived barrier to preventative lifestyle action	Stage of life	Cardiometabolic pregnancy complication of relevance				Stage of life	Postpartum cardiometabolic complication of relevance	
		Pregnancy	GDM	HDP	IUGR	PTB	Postpartum	T2DM	CVD
Opportunity	Lack of support from family (e.g., familial incongruence with healthy eating attempts).	(*n* = 2) [42, 98]	(*n* = 2) [42, 98]	NA	NA	NA	(*n* = 6) [43, 67, 72, 81, 96, 99]	GDM^a^ [43, 67, 72, 96]	HDP^a^ [81, 99]
Opportunity	Lack of support from healthcare professionals (e.g., lack of lifestyle advice, culturally appropriate, and patient‐centered care).	(*n* = 2) [42, 98]	(*n* = 2) [42, 98]	NA	NA	NA	(*n* = 9) [38, 43, 44, 67, 72, 76, 81, 96, 99]	GDM^a^ [43, 67, 72, 76, 96]	HDP^a^ [38, 44, 81, 99]
Opportunity	Social and cultural expectations (e.g., traditional cooking, eating patterns, social eating expectations, and eating within family environments).	(*n* = 4) [42, 87, 92, 98]	(*n* = 4) [42, 87, 92, 98]	NA	NA	NA	(*n* = 2) [66, 72]	GDM^a^ [66, 72]	GDM^a^ [66]
Opportunity	Lack of time due to professional and familial responsibilities.	(*n* = 4) [42, 65, 90, 98]	(*n* = 4) [42, 65, 90, 98]	NA	NA	NA	(*n* = 14) [38, 43, 52, 66, 67, 69, 72, 76, 80, 81, 87, 92, 93, 106]	GDM^a^ [43, 52, 66, 67, 69, 72, 76, 80, 87, 92, 106]	GDM^a^ [66] HDP^a^ [38, 81, 93]
Motivation	Mental health and emotional well‐being (e.g., daily difficulties, physical, mental, emotional stress of self‐managing diet, physical activity, glycemic control, blood glucose monitoring, burden, feeling of obsession and consuming thoughts of management strategies, and postpartum challenges).	(*n* = 2) [98, 108]	(*n* = 2) [98, 108]	NA	NA	NA	(*n* = 2) [92, 96]	GDM^a^ [92, 96]	NA
Motivation	Low motivation.	(*n* = 1) [90]	(*n* = 1) [90]	NA	NA	NA	(*n* = 9) [44, 70, 72, 80, 93, 97, 105, 106, 108]	GDM^a^ [70, 72, 80, 97, 105, 106, 108]	HDP^a^ [44, 93]
Motivation	Low perceived benefit of seeking care and low perception of susceptibility to and severity of cardiometabolic pregnancy complications.	(*n* = 1) [37]	(*n* = 1) [37]	(*n* = 1) [37]	(*n* = 1) [37]	NA	NA	NA	NA
Motivation	Feelings of guilt and selfishness.	NA	NA	NA	NA	NA	(*n* = 4) [38, 80, 87, 106]	GDM^a^ [80, 87, 106]	HDP^a^ [38]
Capability	Exhaustion and fatigue.	NA	NA	NA	NA	NA	(*n* = 6) [38, 67, 69, 80, 87, 92]	GDM^a^ [67, 69, 80, 87, 92]	HDP^a^ [38]
Capability, opportunity	Finances and perception of eating healthier being more expensive.	(*n* = 1) [83]	(*n* = 1) [83]	NA	NA	NA	(*n* = 7) [38, 43, 61, 67, 80, 87, 93]	GDM^a^ [43, 61, 67, 80, 87]	HDP^a^ [38, 93]
Capability, motivation	Difficulties adhering to prescribed lifestyle advice.	NA	NA	NA	NA	NA	(*n* = 4) [53, 70, 77, 78]	GDM^a^ [53, 70, 77, 78]	
Opportunity, motivation	Competing priorities, including prioritizing health of child above own health.	NA	NA	NA	NA	NA	(*n* = 7) [53, 61, 67, 70, 77, 78, 93]	GDM^a^ [53, 61, 67, 70, 77, 78]	HDP^a^ [93]
Capability, opportunity	Having a higher weight, including related stigma and physical challenges of engaging with healthy behaviors.	(*n* = 1) [83]	(*n* = 1) [83]	NA	NA	NA	NA	NA	NA

Abbreviations: CVD: cardiovascular disease, GDM: gestational diabetes mellitus, HDP: hypertensive disorders of pregnancy, IUGR: intrauterine growth restriction, NA: not applicable, PTB: preterm birth, T2DM: type 2 diabetes mellitus.

^a^ Women experienced this cardiometabolic pregnancy complication in a recent pregnancy.

##### 3.2.1.5 Cues to Action

Cues to action refers to internal and external factors that could prompt a health behavior [13, 14].

###### 3.2.1.5.1 Cues to Preventative Action in Diagnosed Women

Among women diagnosed with GDM (*n* = 14) [37, 46, 55, 58, 60, 65, 80, 84, 90, 92, 98, 101, 106, 108], HDP (*n* = 5) [37, 38, 40, 81, 95], and IUGR (*n* = 1) [37], a diagnosis of these complications and knowledge of potential health implications encouraged and motivated women to make lifestyle changes to improve their own and their fetus/baby's health [37, 38, 40, 46, 55, 58, 60, 65, 80, 81, 92, 95, 98, 101, 106, 108]. Greater adherence to lifestyle changes was observed in women with higher levels of social support, higher perceived severity and susceptibility, and a history of pregnancy complications [37, 84, 90].

###### 3.2.1.5.2 Individual, Interpersonal, and Environmental Influences on Risk Perception, Knowledge, and Management

Influences on high‐risk pregnant women's risk perception and management of cardiometabolic pregnancy complications are summarized in Table 3.

**TABLE 3:** Individual, social, and environmental influences impacting on high‐risk pregnant and postpartum women's risk perception, knowledge, and management of cardiometabolic pregnancy complications and future cardiometabolic disease.

Individual, interpersonal, and environmental influences on risk perception, knowledge, and management		Stage of life	Cardiometabolic pregnancy complication of relevance				Stage of life	Postpartum cardiometabolic complication of relevance	
		Pregnancy	GDM	HDP	IUGR	PTB	Postpartum	T2DM	CVD
BMI	No association with risk perception or knowledge.	NA	NA	NA	NA	NA	(*n* = 1) [110]	GDM^a^ [110]	NA
	Higher BMI associated with higher perceived risk and lower BMI associated with lower perceived risk.	(*n* = 2) [41, 82]	(*n* = 1) [82]	(*n* = 1) [82]	NA	(*n* = 1) [41]	(*n* = 5) [53, 70, 74, 77, 78]	GDM^a^ [53, 70, 74, 77, 78]	NA
	Women consider weight as one of multiple unmodifiable/modifiable risk factors when conceptualizing risk.	NA	NA	NA	NA	NA	(*n* = 1) [106]	GDM^a^ [106]	NA
Maternal age	No association with risk perception or knowledge.	(*n* = 1) [41]	NA	NA	NA	(*n* = 1) [41]	(*n* = 2) [74, 104]	GDM^a^ [74, 104] HDP^a^ [104]	GDM^a^ [104] HDP^a^ [104]
	Younger age (30.7 vs. 35.0 years) associated with lower risk perception.	NA	NA	NA	NA	NA	(*n* = 1) [111]	GDM^a^ [111]	NA
Race and ethnicity	No association with risk perception or knowledge.	(*n* = 1) [41]	NA	NA	NA	(*n* = 1) [41]	(*n* = 3) [74, 104, 110]	GDM^a^ [74, 104, 110] HDP^a^ [104]	GDM^a^ [104] HDP^a^ [104]
	Lower risk perception among women from non‐Caucasian versus Caucasian ethnic groups.	NA	NA	NA	NA	NA	(*n* = 1) [78]	GDM^a^ [78]	NA
SES	No association with risk perception or knowledge.	(*n* = 1) [41]	NA	NA	NA	(*n* = 1) [41]	(*n* = 1) [85]	GDM^a^ [85]	NA
	Low household income associated with low‐risk perception.	NA	NA	NA	NA	NA	(*n* = 1) [78]	GDM^a^ [78]	NA
Education level	No association with risk perception.	(*n* = 1) [41]	NA	NA	NA	(*n* = 1) [41]	(*n* = 3) [74, 104, 110]	GDM^a^ [74, 104, 110] HDP^a^ [104]	GDM^a^ [104] HDP^a^ [104]
	Higher education associated with higher knowledge of pregnancy complications.	(*n* = 2) [75, 86]	(*n* = 1) [86]	(*n* = 1) [75]	NA	NA	NA	NA	NA
	Lower education levels associated with lower perceived risk for future cardiometabolic disease.	NA	NA	NA	NA	NA	(*n* = 1) [70]	GDM^a^ [70]	NA
GDM management type	High‐risk perception if managed with insulin versus metformin and/or diet‐controlled.	NA	NA	NA	NA	NA	(*n* = 4) [53, 70, 77, 78]	GDM^a^ [53, 70, 77, 78]	NA
	Higher risk perception increased likelihood of following prescribed GDM diet after birth.	NA	NA	NA	NA	NA	(*n* = 1) [76]	GDM^a^ [76]	NA
Exchanges with healthcare professionals	No difference in future risk perception based on health advice received.	NA	NA	NA	NA	NA	(*n* = 1) [104]	GDM^a^ [104] HDP^a^ [104]	GDM^a^ [104] HDP^a^ [104]
	Main source of information for pregnancy complications, personal risk, risk reduction, and complication management.	(*n* = 4) [37, 65, 90, 94]	(*n* = 3) [37, 65, 90]	(*n* = 2) [37, 94]	(*n* = 1) [37]	NA	NA	NA	NA
	Experiences of lack of consistent, reliable, detailed, timely, and easy to understand information on pregnancy complication risk.	(*n* = 12) [28, 29, 42, 45, 65, 68, 81, 83, 90, 95, 98, 108]	(*n* = 7) [28, 42, 65, 83, 90, 98, 108]	(*n* = 4) [29, 68, 81, 95]	NA	(*n* = 1) [45]	NA	NA	NA
	Psychological impact of and lack of empathy when communicating risk.	(*n* = 2) [29, 98]	NA	(*n* = 2) [29, 98]	NA	NA	(*n* = 1) [79]	GDM^a^ [79]	NA
	Regular engagement associated with better knowledge of pregnancy complications.	(*n* = 2) [70, 75]	(*n* = 1) [70]	(*n* = 1) [75]	NA	NA	(*n* = 1) [104]	GDM^a^ [104] HDP^a^ [104]	GDM^a^ [104] HDP^a^ [104]
	Not all women advised about increased future risk from healthcare professionals.	NA	NA	NA	NA	NA	(*n* = 10) [31, 36, 38, 40, 64, 79, 94, 95, 99, 104]	GDM^a^ [79, 104] HDP^a^ [104]	GDM^a^ [31, 36, 104] HDP^a^ [31, 36, 38, 40, 64, 94, 95, 99, 104] IUGR^a^ [31, 36] PTB^a^ [31, 36]
	Lack of information and/or inconsistent communication on postpartum screening and its significance.	NA	NA	NA	NA	NA	(*n* = 4) [76, 79, 101, 103]	GDM^a^ [76, 79, 101, 103]	NA

	Insufficient postpartum follow‐up amount, timing and content (e.g., addressing future risks, empathetic tailored personal risk information, and risk reduction strategies including lifestyle management and action plans).	NA	NA	NA	NA	NA	(*n* = 17) [31, 32, 36, 40, 43, 44, 47, 52, 53, 61, 79, 81, 87, 93, 97, 99, 108]	GDM^a^ [43, 47, 52, 53, 61, 79, 87, 97, 108]	GDM^a^ [31, 36] HDP^a^ [31, 32, 36, 40, 44, 81, 93, 99] IUGR^a^ [31, 36] PTB^a^ [31, 36]
	Women wanted to be informed about their postpartum risk from healthcare professionals so they could have a chance to act on it.	NA	NA	NA	NA	NA	(*n* = 4) [79, 81, 92, 94]	GDM^a^ [79, 92]	HDP^a^ [81, 94]
	Lifestyle information accessible within community.	NA	NA	NA	NA	NA	(*n* = 1) [67]	GDM^a^ [67]	NA
	More likely to receive information about increased risk of T2DM than CVD.	NA	NA	NA	NA	NA	(*n* = 1) [66]	GDM^a^ [66]	GDM^a^ [66]
Women with prior HDP versus GDM less likely to receive information about increased T2DM and CVD risk.	NA	NA	NA	NA	NA	(*n* = 1) [104]	GDM^a^ [104] HDP^a^ [104]	GDM^a^ [104] HDP^a^ [104]
Social support from partner, family and friends	Benefit of being able to share/learn management strategies (lifestyle, mental, emotional, and pharmacological) from other women with shared lived experience, partners, family, and friends.	(*n* = 5) [37, 43, 83, 90, 98]	(*n* = 4) [37, 43, 83, 98]	(*n* = 2) [37, 90]	(*n* = 1) [37]	NA	NA	NA	NA
	Mental, emotional, physical, and social support is a strong driver in helping better manage pregnancy complications, engage in healthful behaviors, and remain in good health during and after pregnancy.	(*n* = 6) [33, 42, 43, 49, 65, 87]	(*n* = 6) [33, 42, 43, 49, 65, 87]	NA	NA	NA	(*n* = 7) [28, 38, 80, 81, 92, 99, 103]	GDM^a^ [33, 97, 103]	HDP^a^
	Women sought opinions from their social support network to better understand personal risk and prevention strategies.	(*n* = 1) [83]	(*n* = 1) [83]	NA	NA	NA	(*n* = 3) [33, 97, 103]	GDM^a^	NA
	Women suggested that support networks of women with shared lived experience could reduce isolation and increase engagement in healthful behaviors postpartum.	NA	NA	NA	NA	NA	(*n* = 3) [95, 99, 103]	GDM^a^ [103]	HDP^a^ [95, 99]
Family history of cardiometabolic disease	Associated with greater knowledge of family history as a risk factor for cardiometabolic pregnancy complications.	(*n* = 4) [33, 46, 57, 108]	(*n* = 4) [33, 46, 57, 108]	NA	NA	NA	NA	NA	NA
	Family history associated with moderate or high perceived risk and no family history with low perceived risk.	(*n* = 2) [41, 98]	(*n* = 1) [98]	NA	NA	(*n* = 1) [41]	(*n* = 16) [38, 52, 53, 66, 67, 70, 73, 74, 77, 78, 81, 96, 101, 103, 106, 110]	GDM^a^ [52, 53, 66, 67, 70, 73, 74, 77, 78, 96, 101, 103, 106, 110]	GDM^a^ [66] HDP^a^ [38, 81]
	Increased awareness for engagement in postpartum screening.	NA	NA	NA	NA	NA	(*n* = 2) [101, 103]	GDM^a^ [101, 103]	NA
	Greater knowledge of future cardiometabolic disease risk and interest in preventative behaviors.	NA	NA	NA	NA	NA	(*n* = 1) [96]	GDM^a^ [96]	NA
	Contributed to belief that future cardiometabolic disease is inevitable, stress and fear of future diagnosis, avoidance of preventative behaviors, and/or normalizing of cardiometabolic disease, resulting in a lack of perceived severity.	NA	NA	NA	NA	NA	(*n* = 3) [72, 87, 92]	GDM^a^ [72, 87, 92]	NA
Internet and online applications	Used to gain knowledge of pregnancy complications and future risks, especially when motivated to reduce future risk.	(*n* = 4) [37, 83, 90, 95]	(*n* = 3) [37, 83, 90]	(*n* = 2) [37, 95]	(*n* = 1) [37]	NA	(*n* = 10) [32, 36, 40, 44, 64, 94, 95, 97, 99, 103]	GDM^a^ [97, 103]	GDM^a^ [31, 36] HDP^a^ [32, 36, 40, 44, 64, 94, 95, 99] IUGR^a^ [31, 36] PTB^a^ [31, 36]

Abbreviations: BMI: body mass index, CVD: cardiovascular disease, GDM: gestational diabetes mellitus, HDP: hypertensive disorders of pregnancy, IUGR: intrauterine growth restriction, NA: not applicable, PTB: preterm birth, SES: socioeconomic status, T2DM: type 2 diabetes mellitus.

^a^ Women experienced this cardiometabolic pregnancy complication in a recent pregnancy.

##### 3.2.1.6 Self‐Efficacy

Self‐efficacy refers to one's beliefs in their own ability to perform a recommended health behavior [13, 14]. One study suggested that women with GDM thought they could change their risk (64%) and were optimistic and confident that lifestyle changes (96%) and medical therapy (89%) could improve their management of GDM [51]. Women at risk of GDM, HDP, or IUGR believed they could lower their risk and/or avoid experiencing a cardiometabolic complication by engaging in self‐care (healthy diet, exercise, weight management, regular check‐ups, not drinking, not smoking, and pregnancy planning) [37]. This belief persisted in the presence of a history of pregnancy complications and a family history of cardiometabolic disease for women at risk of HDP [38], although it did not always correlate with taking action (in this instance for women at risk of GDM) [83].

#### 3.2.2 High‐Risk Postpartum Women

##### 3.2.2.1 Perceived Susceptibility

###### 3.2.2.1.1 Perceived Susceptibility to Cardiometabolic Disease Following GDM, HDP, IUGR, and PTB

Most [28, 33, 46, 48, 52, 55, 57, 61, 63, 66, 70, 72, 73, 78, 79, 83, 86, 91, 92, 97, 101, 103, 105, 106, 108, 111], but not all studies [42, 57, 58, 71, 76, 87, 89, 90, 98, 102, 110], reported the majority of women with prior GDM were aware it is a risk factor for T2DM. However, women were unaware of their exact risk for future T2DM or perceived their likelihood of developing T2DM as low to moderate [28, 33, 35, 39, 46, 47, 48, 49, 50, 51, 52, 53, 54, 55, 57, 58, 60, 63, 66, 67, 69, 70, 73, 74, 76, 77, 78, 79, 80, 85, 87, 89, 92, 96, 97, 98, 102, 103, 104, 106, 107, 108, 110, 111]. Very few women perceived themselves to be high risk for developing future T2DM, varying from none or almost no risk [53, 54, 97, 110], or when asked to quantify their risk, 16%–90% perceived themselves as high risk [39, 47, 53, 54, 70, 77, 78, 85, 91, 104, 106, 110, 111]. One study reported higher risk perception for T2DM was attributed to a family history of diabetes, having a higher weight, and poor lifestyle rather than a history of GDM [90]. In the more limited literature for CVD, some but not all [38, 86] studies reported the majority of women were aware GDM is a risk factor for future CVD [31, 36, 71] and perceived their future susceptibility as moderate or high [36, 60, 66, 67, 104]. Where quantified, 57.9% of women with previous GDM perceived their risk of future CVD as high [36].

Some [36, 40, 44, 64, 94, 95, 109] but not all [29, 31, 32, 81, 93, 99] studies reported women were aware HDP is a risk factor for future CVD. Knowledge of future risk was more accurate in women with a history of HDP in prior pregnancies [29] and in women who had regular medical assessments (blood pressure, glucose, cholesterol, and renal function) and were taking antihypertensive medications [32]. Where quantified, 25.0%–36.4% of women perceived themselves as high risk for T2DM following HDP [94, 104]. Perceived susceptibility for CVD following HDP was a variable across low, moderate, and high, with personal risk not well understood by many women [36, 38, 44, 81, 94, 104, 109]. Where quantified, 55.1% of women believed themselves to be high risk for future CVD [36], 69.0% for future heart attack, 64.0% for future stroke, 67.0% for future heart disease, 50.0% for future peripheral vascular disease, and 76.0% for future chronic hypertension [94] following HDP. Two studies reported 12.5%–20.0% of women with prior IUGR and 0%–15.4% of women with prior PTB were aware of their risk of future CVD [31, 36]. Other women with IUGR (37.6%) and PTB (34.6%) perceived themselves as high risk for future CVD [36].

###### 3.2.2.1.2 Distinction Between Knowledge of Risk and Perception of Risk

Knowledge of GDM [28, 36, 79, 110], HDP [36, 44], IUGR [36], and PTB [36] as risk factors for future cardiometabolic disease was distinct from risk perception, occasionally leading to a higher risk perception [36, 110] but not always [28, 44, 79].

###### 3.2.2.1.3 Knowledge of Risk Factors for Future Cardiometabolic Disease

Many studies reported on high‐risk postpartum women's knowledge of risk factors for cardiometabolic disease, including women who had experienced GDM [36, 51, 52, 66, 69, 70, 90, 106, 111], HDP [36, 81, 109], IUGR [36], and PTB [36, 109]. Genetics and family history [52, 66, 90, 106], sedentary behavior [36, 66, 81, 90], race and ethnicity [66], poor weight management [36, 51, 66, 90, 106], suboptimal diet [52, 66, 81, 90], stress [66, 81], smoking [36, 66, 109], hypertension [36], high cholesterol [36, 109], and aging [51] were risk factors known to increase T2DM and CVD risk. Overall, knowledge of risk factors among women with prior GDM was low [111] to moderate [69, 70]. Greater knowledge of risk factors was reported in women who perceived their risk of developing future cardiometabolic disease as moderate to high compared with women who perceived their risk as low or none [70].

###### 3.2.2.1.4 Knowledge of Screening for Future Cardiometabolic Disease

Knowledge of screening for future cardiometabolic disease following GDM was low in the majority of studies [57, 73, 76, 79, 101], with one exception [103]. Women were unaware of screening importance and recommendations [76, 79, 101], the need for ongoing screening beyond the first screen [73] or were uncertain when to arrange screening tests [57].

###### 3.2.2.1.5 Knowledge of Postpartum Preventative Lifestyle Behaviors

Women's knowledge of cardiometabolic preventive lifestyle behaviors following GDM [28, 30, 33, 36, 46, 48, 51, 52, 61, 62, 66, 67, 69, 71, 72, 73, 86, 97, 102, 103, 105, 106, 111], HDP [36, 81, 92, 109], IUGR [36], and PTB [36, 109] was a variable. Women were aware that consuming a healthy diet [28, 33, 36, 46, 48, 51, 52, 61, 62, 66, 67, 72, 73, 81, 92, 103, 105, 106, 111], engaging in regular physical activity [28, 33, 36, 46, 48, 51, 52, 61, 62, 66, 67, 72, 73, 81, 92, 103, 105, 106, 111], maintaining a healthy weight [33, 36, 46, 51, 52, 66, 92, 103, 105, 106, 111], not smoking [36, 67], managing stress [61, 81], maintaining good mental health [61, 62], engaging in breastfeeding [36, 71, 86], and medical therapy [51] would reduce their risk of future cardiometabolic disease. Some studies reported overall knowledge of preventive strategies as poor [30, 48, 97, 109] to moderate [36, 69]. While some women commented on postpartum lifestyle behaviors they thought they should be doing (diet, physical activity, and breastfeeding) for general health, they were not aware of the impact of these changes on future cardiometabolic disease risk [28, 52, 73, 102].

##### 3.2.2.2 Perceived Severity

There was a distinct postpartum emotional impact of experiencing GDM [43, 47, 48, 52, 61, 62, 79, 87, 96, 101, 102, 103, 108] and HDP [38, 44, 81, 93, 95, 99]. Postpartum women reported feeling abandoned by the healthcare system due to a lack of follow‐up after birth and a lack of encouragement, interest in their health, and tailored information regarding personal risk of future disease and management strategies from healthcare providers [43, 52, 99, 108]. Women felt worry and concern [38, 44, 47, 48, 61, 62, 79, 81, 93, 96, 108], fear [61, 79, 95, 101, 103], and anxiety related to the potential of future cardiometabolic disease [79, 81]. Women with a family history of cardiometabolic disease often felt powerless in their ability to reduce their future risk [102, 108]. Guilt, shame, and embarrassment associated with a diagnosis often continued through to the postpartum period, where the emotional impact of experiencing a pregnancy complication negatively affected their postpartum health‐related behaviors [79, 87, 108]. Some women felt surprise when they first gained knowledge of the link between their pregnancy complication and future cardiometabolic disease [44, 81] while others felt motivated and empowered to reduce their risk [95, 96].

##### 3.2.2.3 Perceived Benefits

Perceived benefits of engaging in healthy practices post GDM [33, 36, 48, 51, 52, 66, 67, 73, 92, 103, 105, 106, 111], HDP [36, 38, 81, 93], IUGR [36], and PTB [36] included reduced risk of future cardiometabolic disease; improved physical, mental, and social health; weight management; longevity; and the ability to stay healthy to care for family and act as a good role model.

##### 3.2.2.4 Perceived Barriers

###### 3.2.2.4.1 Barriers to Engaging in Postpartum Preventative Lifestyle Action

Barriers to preventative lifestyle action following GDM [43, 52, 53, 67, 70, 72, 97, 105, 108] and HDP [44, 81] were present. Several studies reported women having no or low adherence to a healthy lifestyle postpartum [44, 70, 72, 97, 105, 108] while others reported women had attempted but were not sustaining healthy lifestyle changes [43, 52, 53, 67, 72, 81, 105]. Commonly reported barriers to adopting a healthy lifestyle according to core constructs within the COM‐B model are summarized in Table 2.

###### 3.2.2.4.2 Barriers to Engaging in Postpartum Screening

Various barriers to engaging in postpartum screening following GDM [35, 72, 76, 79, 90, 97, 101, 103] and HDP [38, 44] were acknowledged. Limited awareness of the need for screening, inadequate communication by healthcare professionals, lack of time, lack of social support, unpleasant and inconvenient testing (oral glucose tolerance test, fasting, needles, length of time, length of travel, and child caring arrangements), fear, uncertainty, anxiety, shame, and guilt associated with a potential cardiometabolic disease diagnosis [35, 38, 44, 72, 76, 79, 90, 97, 101, 103]. Additional barriers included low‐risk perception for future cardiometabolic disease, the belief that maintaining healthy behavioral changes mitigated any future risks, prioritizing the child's health over one's own health, forgetting to attend, an unpleasant delivery experience, health issues in a newborn baby, and postpartum adjustment to a newborn baby [35, 76, 79, 90, 101, 103].

##### 3.2.2.5 Cues to Action

###### 3.2.2.5.1 Individual, Interpersonal, and Environmental Influences on Postpartum Risk Perception, Knowledge, and Management

The social, physical, and environmental influences on risk perception and knowledge following a cardiometabolic pregnancy complication are summarized in Table 3.

###### 3.2.2.5.2 Facilitators to Postpartum Preventative Action

Among studies in women with prior GDM (*n* = 19) [33, 46, 48, 55, 61, 63, 67, 69, 70, 72, 76, 87, 92, 96, 97, 98, 103, 105, 108] and HDP (*n* = 7) [38, 40, 44, 81, 93, 95, 99], many reported that the majority of women maintained a healthy lifestyle postpartum [44, 46, 48, 55, 61, 72, 76, 93, 98, 99, 105]. Facilitators to postpartum preventative action included women's motivation to be a good role model and improve the health of their family, including reducing future chronic disease risk in their family [38, 40, 61, 67, 81, 92, 93, 95, 96], improving their own general health, and reducing their own long‐term risk of cardiometabolic disease and risks during subsequent pregnancies [33, 40, 44, 48, 55, 61, 67, 76, 81, 87, 92, 93, 96, 97, 103, 105]. Other facilitators to healthy lifestyle behaviors included monitoring health behaviors, planning, goal setting, accountability, flexibility, seeing the benefits of their achievements, implementing sustainable changes, social support from others, positive thinking, having more follow‐ups, and receiving ample lifestyle advice from healthcare professionals [44, 46, 48, 55, 61, 72, 76, 93, 98, 99, 105]. Several studies reported on the link between risk perception and adherence to a healthy lifestyle, where low‐risk perception for future cardiometabolic disease was associated with low adherence and the converse [63, 69, 76, 87, 108], although this was not consistently reported [48, 87]. One study reported women who perceived themselves as moderate or high risk more often demonstrated greater planning and intention to modify their future lifestyle behaviors compared with women with lower risk perceptions [70].

###### 3.2.2.5.3 Facilitators to Engagement in Postpartum Screening

Engagement in postpartum cardiometabolic screening following GDM [53, 85, 90, 104] and HDP [40, 53, 64, 104] was low [90] in some but not all studies, with 73.0%–95.7% of women reporting attendance where quantified [40, 53, 64, 85, 104]. Facilitators to screening attendance following GDM were awareness of importance, family history of cardiometabolic disease, self‐perceived high risk for future cardiometabolic disease, adequate emotional and social support, access to caregivers, perception of screening as obligatory, screening reminders, prior experience of obstetric complications, and general practitioner knowledge, initiative, and viewpoint [79, 101, 103].

##### 3.2.2.6 Self‐Efficacy

###### 3.2.2.6.1 Beliefs About Self‐Ability to Reduce Future Cardiometabolic Risk

Some women with or previously diagnosed with GDM [55, 63, 66, 67, 73, 92, 97, 105] and HDP [38, 44, 81, 93] viewed their future cardiometabolic risk as largely out of their control; felt they lacked the necessary skills, tools, and knowledge to reduce their risk; or thought they had no influence over their personal risk postpartum. Conversely, some women with or previously diagnosed with GDM [48, 51, 55, 61, 66, 90, 92, 104, 105] and HDP [44, 81, 104] were confident in their ability and intended to engage in regular physical activity and consume a healthy diet postpartum. In some studies, a low‐risk perception for cardiometabolic disease following GDM led to higher optimism about not developing future cardiometabolic disease, and a high‐risk perception led to lower optimism [67, 70, 90, 111].

#### 3.2.3 Summary of Key Findings

**FIGURE 3:**
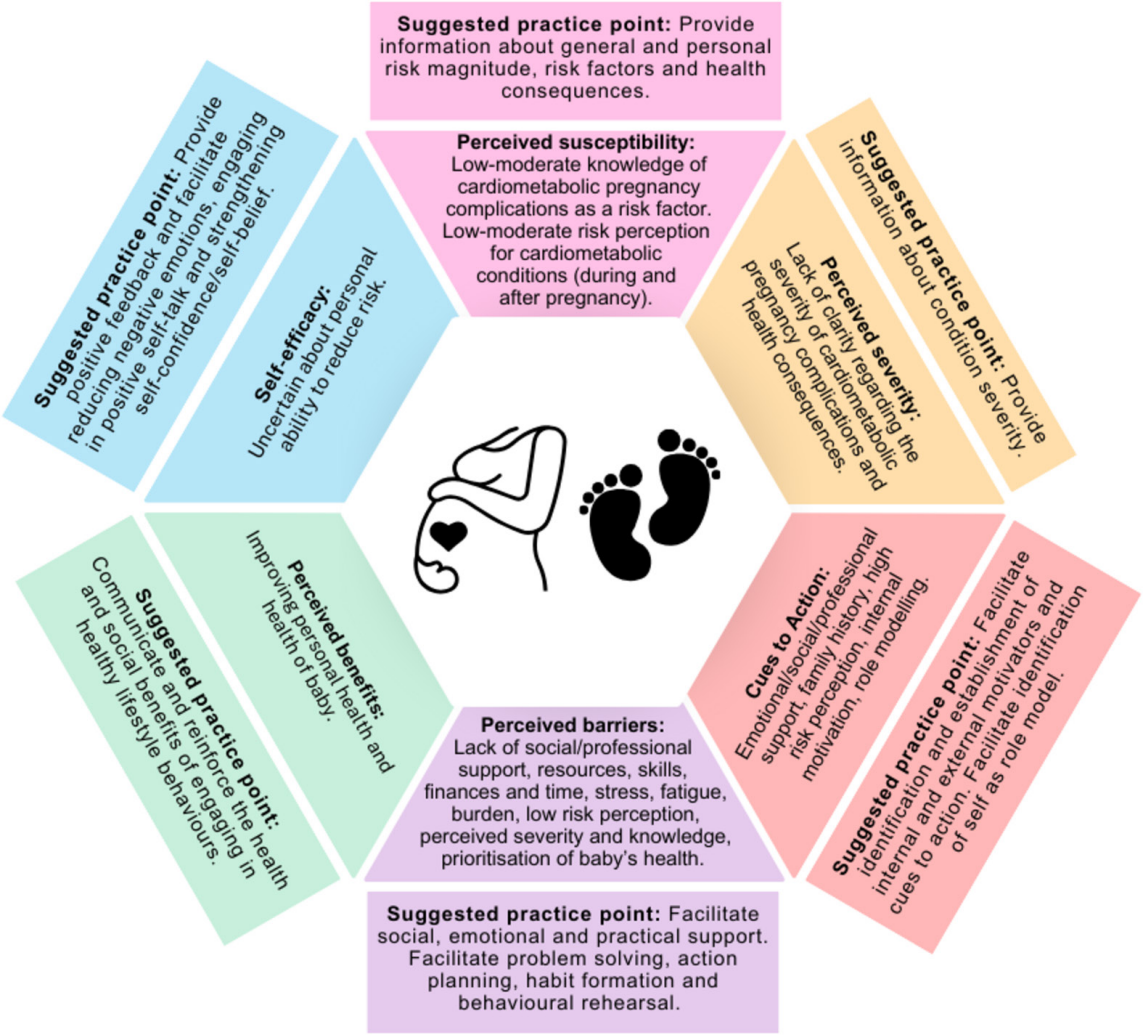
Summary of key findings according to the HBM and key practice recommendations to improve high‐risk pregnant and postpartum women's risk perception and management of cardiometabolic pregnancy complications and postpartum cardiometabolic disease.

Key research findings according to the HBM were used to generate recommendations for practice/interventions that aim to optimize women's risk perception using the BCW (Figure 3). Relevant BCTs that can be used to optimize women's risk perception were identified (Table 4), with a detailed breakdown of how these BCTs were selected shown in Table S5.

**TABLE 4:** Summary of intervention strategies for optimizing risk perception and management of cardiometabolic pregnancy complications and future cardiometabolic disease among high‐risk pregnant and postpartum women.

HBM construct	Behavior change techniques [19, 20]		Synthesized examples of proposed intervention strategies
	Number	Name	
Perceived susceptibility	5.1	Information about health consequences	Provide education and information about general and personal risk, risk factors, and health consequences.
	5.3	Information about social and environmental consequences	Provide information on risks, implications, and the benefits of reducing their risk for their families.
	7.1	Prompts/cues	Risk education to be used as a cue to action for lifestyle engagement.
	9.1	Credible source	Accredited healthcare professional to provide education.
Perceived severity	5.1	Information about health consequences	Provide education and information about general and personal risk, risk factors, health consequences, and condition severity.
	5.3	Information about social and environmental consequences	Provide information on condition severity and the benefits of reducing their risk for their families.
	7.1	Prompts/cues	Condition severity education to be used as a cue to action for lifestyle engagement.
	9.1	Credible source	Accredited healthcare professional to provide education.
Perceived barriers	1.2	Problem solving	Encourage women to identify potential barriers and facilitators to lifestyle change and ways to overcome barriers.
	1.4	Action planning	Assist women in creating a plan on how to achieve their SMART goals. Prompt planning the performance of a particular health behavior.
	3.1/3.3	Social support (unspecified)/social support (emotional)	Arrange for social support(s) to provide verbal encouragement, positive words of affirmation, emotional and moral support.
	3.2	Social support (practical)	Encourage women to speak to their social support(s) about their lifestyle related SMART goal(s) and how and when social support(s) can assist. Refer women to appropriate community groups.
	4.1	Instruction on how to perform the behavior	Instruct women on how to eat healthy, prepare meals, and snacks and exercise in a manner conducive to reducing cardiometabolic disease risk during and after pregnancy.
	5.1	Information about health consequences	Provide education and information about general and personal risk, risk factors and health consequences. Provide education and information on benefits of engaging in a healthy lifestyle and regular cardiometabolic screening.
	6.1	Demonstration of the behavior	Provide visual, audio, and audio‐visual information provide practical examples and demonstrations of how to engage with health promoting behaviors.
	7.1	Prompts/cues	Encourage women to introduce or define environmental or social stimulus for the purpose of prompting or cueing positive self‐talk and healthful behaviors.
	8.1	Behavioral practice/rehearsal	Encourage women to practice and continue healthy behaviors.

	8.3	Habit formation	Prompt rehearsal and repetition of behaviors that overcome certain barriers to engaging in healthful behaviors.
	9.1	Credible source	Accredited healthcare professional to provide education.
	11.2	Reduce negative emotions	Advise women on the use of stress management skills (e.g., meditation, deep breathing, journaling, and getting adequate sleep) to help reduce mental and emotional stress.
	12.1	Restructuring the physical environment	Help women to identify changes in their physical environment that can be made to assist with achieving their behavioral SMART goal(s) to reduce cardiometabolic risk.
	12.2	Restructuring the social environment	Advise women to minimize time spent with individuals in their social environment who engage in behaviors that contraindicate living a healthy lifestyle and increase time spent with those living a healthy lifestyle.
12.5	Adding objects to the environment	Encourage women to add objects to their environment in order to facilitate behavioral change.
Self‐efficacy	2.2	Feedback on behavior	Review and provide positive/practical feedback on optimizing healthy lifestyle behaviors to increase women's confidence.
	2.7	Feedback of outcome(s) of behavior	Provide feedback on the outcome of the performance of health behaviors to help demonstrate to women how their behavior may be contributing to reduced cardiometabolic risk.
	3.1	Social support (unspecified)	Arrange for social support(s) to provide verbal encouragement, positive words of affirmation, and emotional and moral support.
	3.3	Social support (emotional)	Arrange for social support(s) to provide verbal encouragement, positive words of affirmation, and emotional and moral support.
	5.1	Information about health consequences	Provide education and information about general and personal risk, risk factors, and health consequences. Provide education and information on benefits of engaging in a healthy lifestyle and regular cardiometabolic screening.
	6.2	Social comparison	Provide examples of real women and/or data relating to the effect of lifestyle change in reducing cardiometabolic disease risk during and after a complicated pregnancy as an exemplar for women.
	7.1	Prompts/cues	Encourage women to introduce or define environmental or social stimulus for the purpose of prompting or cueing positive self‐talk and self‐belief.
	9.1	Credible source	Accredited healthcare professional to provide education.

	11.2	Reduce negative emotions	Advise women on ways of reducing negative emotions, including self‐doubt, stress, and anxiety associated with being labeled as high risk for cardiometabolic disease development.
	15.1	Verbal persuasion about capability	Encourage and inform women that they can successfully reduce their cardiometabolic disease risk during and after pregnancy by engaging in a healthy lifestyle, arguing against self‐doubts and assuring them that they can and will succeed.
15.4	Self‐talk	Facilitate positive self‐talk, either aloud and/or inside own head to increase women's confidence and self‐belief.
Cues to action	3.1	Social support (unspecified)	Encourage social support(s) to provide advice, trigger, or enable the decision‐making process to initiate recommended health actions.
	3.3	Social support (emotional)	Encourage social support(s) to provide advice, trigger, or enable the decision‐making process to initiate recommended health actions.
	5.1	Information about health consequences	Provide education and information about general and personal risk, risk factors, health consequences, and condition severity.
	5.3	Information about social and environmental consequences	Provide information on risks, implications, condition severity, and the benefits of reducing their risk for their families.
	7.1	Prompts/cues	Risk education to be used as a cue to action for lifestyle engagement.
	9.1	Credible source	Accredited healthcare professional to provide education.
	12.5	Adding objects to the environment	Provide flyers and posters to be displayed in GP clinics, hospitals, and community centers that educate women about risk factors, health consequences, screening, and benefits of living a healthy lifestyle.
	13.1	Identification of self as role model	Utilize women's desire to be a good role model for their children and other family members as a stimulus to trigger health action.
Perceived benefits	2.4	Self‐monitoring of outcome(s) of behavior	Establish a method for women to monitor and record the outcomes of their health behaviors to reinforce benefits.
	2.7	Feedback of outcome(s) of behavior	Monitor and provide feedback on the outcome of the performance of health behaviors and the observed benefits to health.
	5.1	Information about health consequences	Provide education and information about general and personal risk, risk factors, health consequences, and condition severity. Provide education and information on benefits of engaging in a healthy lifestyle and regular cardiometabolic screening.
	5.3	Information about social and environmental consequences	Provide information on risks, implications, condition severity, and the benefits of reducing their risk for their families.
	7.1	Prompts/cues	Risk education to be used as a cue to action for lifestyle engagement.
	9.1	Credible source	Accredited healthcare professional to provide education.
	13.1	Identification of self as role model	Utilize women's desire to be a good role model for their children and other family members as a stimulus to trigger health action.

Abbreviations: HBM: health belief model, SMART: specific, measurable, achievable, realistic, time‐bound.

## 4 Discussion

For the first time, we systematically investigate how risk perception influences high‐risk women across various cardiometabolic conditions during and after pregnancy. Risk perception was individual, context‐specific, and variable across life stages and conditions, with more known about GDM and HDP than IUGR and PTB. Risk perception and women's ability to act on their perceived risk through preventative lifestyle and management strategies was associated with various internal and external factors. Adequate knowledge of cardiometabolic pregnancy complications improved pregnant women's risk perceptions. Knowledge of cardiometabolic pregnancy complications as risk factors for future cardiometabolic disease led to higher risk perceptions for these, which positively influenced adherence to preventative lifestyle behaviors.

### 4.1 Low‐Risk Perception Among Women

Many high‐risk pregnant and postpartum women did not perceive their risk as high. Knowledge of cardiometabolic pregnancy complications and future diseases (across knowledge of risk factors, perceived severity, symptoms, management, and health consequences) was a key domain influencing risk perception [30, 34, 36, 70, 110]. This is consistent with general population research for risk factors including education, BMI, and family history [112, 113, 114]. Our findings reinforce that risk perception is influenced by a multitude of factors and cannot be generalized based on isolated characteristics or cardiometabolic pregnancy complications alone. We note that the highest risk perceptions for future T2DM or CVD were among women who have previously experienced GDM or HDP, respectively. Given the limited research outside of GDM, it is still unclear whether this is related to the cardiometabolic pregnancy complication experienced or women's knowledge of other sociodemographic, anthropometric, lifestyle, or clinical cardiometabolic risk factors (e.g., glucose vs. blood pressure). Only one included study made this distinction, reporting that high‐risk perception for future T2DM following GDM was attributed to a family history of T2DM, having a higher weight, and poor lifestyle rather than having GDM [90]. Individual assessments of risk perception are therefore necessary to understand and optimize risk perception among high‐risk women during and following pregnancy. With no reports on risk perceptions for T2DM following IUGR and PTB and limited reports for CVD, further exploration of risk perception in women at risk of or diagnosed with IUGR and PTB is also warranted.

### 4.2 Distinction Between Knowledge and Risk Perception and the Knowledge–Behavior Gap

We found, in addition to knowledge, high‐risk pregnant and postpartum women require skills, motivation, self‐efficacy, the perception that they can control and influence their risk, alongside social and professional support to successfully implement lifestyle change. Knowledge of risk for cardiometabolic pregnancy complications and cardiometabolic disease is considered a prerequisite for preventative lifestyle action [115]. However, we report that knowledge of risk and risk perception can be distinct. While women may be aware of their increased risk and be knowledgeable about risk factors, this does not necessarily mean they perceive their risk as high. Knowledge is more likely to influence risk perception when coupled with internal and external influences and beliefs and attitudes about potential harm to make a self‐assessment about the risk of an event and its consequences [116, 117, 118]. However, existing research notably associates risk perception, as opposed to knowledge, as central to behavior change [13, 14, 15]. Crucially, it is not enough to improve knowledge alone to achieve behavior change in these high‐risk women.

### 4.3 Inadequate Risk Communication by Healthcare Professionals

In the general population, being informed about individual cardiometabolic disease risk increases a person's risk perception [119]. Crucially, we note that inadequate risk communication by some healthcare professionals contributed to poor risk perception and knowledge in some women [28, 29, 36, 38, 40, 42, 45, 64, 65, 66, 68, 76, 79, 81, 83, 90, 94, 95, 98, 99, 101, 103, 104, 108]. There is a well‐documented existing need in this population group for more in‐depth education including defining complications, likely time of complication onset, modifiable and nonmodifiable risk factors, preventative measures, means of diagnosis, identifying and managing symptoms, short‐term and long‐term health consequences for mother and baby, and management strategies [120, 121, 122, 123, 124, 125, 126, 127, 128, 129]. Adding to this, we found inadequate risk communication extended beyond a lack of in‐depth communication of cardiometabolic pregnancy complications and personal risk to a lack of patient‐centered preventative lifestyle advice, management strategies and follow‐up, alongside the unempathetic, unsupportive, and disempowering nature in which risk is commonly communicated [29, 32, 36, 40, 43, 44, 47, 52, 53, 61, 79, 81, 87, 93, 97, 98, 99, 108]. We also report being diagnosed with and experiencing a cardiometabolic pregnancy complication can negatively impact women's mental and emotional well‐being during pregnancy and postpartum [28, 33, 38, 44, 46, 47, 48, 49, 50, 57, 58, 60, 61, 62, 72, 79, 81, 84, 87, 90, 92, 93, 95, 96, 98, 101, 102, 103, 108]. The literature suggests, when coupled with suboptimal risk communication by healthcare professionals, this may exacerbate feelings of being unsupported. This can result in women being less likely to seek care, trust, and take on healthcare professional advice, less likely to act on their risk, as well as confusion and anxiety about how to appropriately act on their risk [130, 131, 132].

### 4.4 Looking Towards Improving Cardiometabolic Risk Perceptions in High‐Risk Pregnant and Postpartum Women

Our findings highlight that it is imperative to communicate personal risk to all high‐risk women in an empowering, supportive, and empathetic manner that respects lived experience and improves their risk perception. Communication must be followed up with a patient‐centered, ongoing, and collaborative approach to risk prevention and lifestyle management to improve cardiometabolic risk perceptions [133]. Existing research suggests that for these high‐risk women [120, 121, 122, 123, 124, 125, 126, 127, 128, 129] and the general population [133, 134, 135], understanding of cardiometabolic risk, risk perception, and taking preventative action are enhanced by incorporating the following approaches into risk education: provision of risk information by an accredited healthcare professional, use of multiple delivery modes (e.g., verbal, written, visual, interactive, wed‐based, paper‐based, and text messages), patient‐centered care, facilitation of behavior change skill development (e.g., goal setting and self‐monitoring), encouragement of help seeking (social, professional, and peer support), and use of a shared decision aid that facilitates joint decision‐making on management strategies [120, 121, 122, 123, 124, 125, 126, 127, 128, 129, 133, 134, 135]. This will likely improve risk perception. We recommend these approaches be taken when engaging high‐risk pregnant and postpartum women for education on cardiometabolic pregnancy complications and future cardiometabolic disease. It is also paramount for healthcare professionals to understand the complexities of the pregnancy and postpartum period, including new responsibilities and the changes and challenges women face in addition to managing a cardiometabolic pregnancy complication diagnosis. Additionally, we acknowledge that alongside personal barriers to optimizing risk perception and behavior change, at a healthcare system level, the organizational and funding structure of publicly available pregnancy and postpartum services for these women presents additional barriers to healthcare access, utilization, and benefit [136, 137, 138, 139]. These barriers can include long wait times, inflexible scheduling, short consultation times, lack of continuity of care, poor communication, language and cultural barriers, discrimination, lack of infrastructure, and issues with transition of care (e.g., from the hospital during pregnancy to primary care postpartum) [136, 137, 138, 139]. Interventions to address these barriers, in addition to personal barriers to optimizing risk perception and engagement with preventative health behaviors, are required.

### 4.5 Strengths and Limitations

This review extends previous research by assessing cardiometabolic risk perception among women beyond just those at high risk of or who have experienced GDM and HDP, to include women at risk of or who have experienced IUGR and PTB. While GDM, HDP, IUGR, and PTB have distinct pathophysiological processes leading to suboptimal maternal and/or fetal outcomes, the metabolic stress and vascular changes of pregnancy that predispose patients to these conditions underpins a shared risk for future cardiometabolic complications [140]. Additionally, while IUGR and PTB describe fetal outcomes with complex etiologies, they are often a consequence of maternal pathophysiological process such as GDM and HDP [140]. This strengthens the need to include IUGR and PTB alongside GDM and HDP as sex‐specific risk factors for cardiometabolic conditions and a further opportunity for education and prevention.

Additionally, the use of a theory‐based approach to data analysis and synthesis (HBM, COM‐B, TDF, and BCW) strengthens the research findings by providing robust methods for data collection, analysis, and synthesis, allowing the development of a more comprehensive understanding of factors influencing health attitudes and behaviors to support improved design and success of behavior change interventions [18, 141, 142, 143]. Theoretical triangulation (combining two or more theories in a research project) allowed for a more comprehensive analysis of the research question(s) and increased the validity of findings generated [144, 145]. While there were varied sociocultural backgrounds included in the review, a deeper understanding of the differences that exist in risk perceptions based on race and ethnicity, SES, area of residence, health status, education, and income level is warranted to better inform interventions to enhance cardiometabolic risk perceptions in high‐risk pregnant and postpartum women.

## 5 Conclusion

This review highlights that high‐risk pregnant and postpartum women's risk perceptions for cardiometabolic pregnancy complications and future cardiometabolic disease are mixed, unique to each woman, and influenced by a variety of internal and external factors. Awareness of risk did not always translate to accurate risk perception, and there is a need to move beyond simple knowledge provision to optimize risk perception and support behavior change. This review emphasizes the importance for high‐risk women to form accurate cardiometabolic disease risk perceptions during pregnancy and postpartum to support informed health decision‐making and engagement in preventative lifestyle behaviors. Consideration of broad contextual factors is required when providing pregnancy and postpartum interventions that aim to improve risk perception among these high‐risk women.

## Conflicts of Interest

The authors declare no conflicts of interest.

## Supporting information


**Table S1.** Search strategy for electronic databases*.
**Figure S1.** The constructs of the capability, opportunity, and motivation model of behavior change (COM‐B) that were integrated into the health belief model (HBM) to synthesize the findings.
**Table S2.** Quality assessment of included qualitative studies (*n* = 39).
**Table S3.** Quality assessment of included quantitative studies (*n* = 38).
**Table S4.** Quality assessment of included mixed‐methods studies (*n* = 7).
**Table S5.** Interventions strategies for optimizing risk perception and management of cardiometabolic pregnancy complications and future cardiometabolic disease among high‐risk pregnant and postpartum women.
