# Supplementary material for: What Is My Risk? A Mixed‐Methods Systematic Review of Risk Perception for Cardiometabolic Pregnancy Complications and Future Cardiometabolic Disease Development
Source: Obes Rev. 2025 Jul 9;26(11):e13967. doi: 10.1111/obr.13967 (PMC12531758; doi:10.1111/obr.13967)
Supplement: Supplementary file 1 — Table S1. Search strategy for electronic databases*. Figure S1. The constructs of the capability, opportunity, and motivation model of behavior change (COM‐B) that were integrated into the health belief model (HBM) to synthesize the findings. Table S2. Quality assessment of included qualitative studies (n = 39). Table S3. Quality assessment of included quantitative studies (n = 38). Table S4. Quality assessment of included mixed‐methods studies (n = 7). Table S5. Interventions strategies for optimizing risk perception and management of cardiometabolic pregnancy complications and future cardiometabolic disease among high‐risk pregnant and postpartum women. [file OBR-26-e13967-s001.pdf]

TITLE

What is my risk? A mixed-methods systematic review of risk perception for cardiometabolic pregnancy complications and future cardiometabolic disease development.

AUTHORS

Elaine K Osei-Safo[1], Jennifer McIntosh[2], Shakira Onwuka[2], Sophia Torkel[1], Margaret McGowan[1], Kristie Cocotis[3], Caitlyn Angel[1], Sanjay Varatharaj[1], Helena Teede[1], Angela Melder[4], Sarah Lang[1], Lisa Moran[1]

AFFILIATIONS

[1]Monash Centre for Health Research and Implementation, School of Clinical Sciences, Monash University, Victoria, Australia.

[2]Melbourne School of Population and Global Health, The University of Melbourne, Carlton, VIC, AUS

[3]Diabetes Victoria, Carlton, VIC, AUS

[4]Health and Social Care Unit, Monash University, Clayton, VIC, AUS

CORRESPONDING AUTHOR

Elaine Osei-Safo
Level 1, 43-51 Kanooka Grove
Clayton VIC 3168
Australia
Email: elaine.osei-safo@monash.edu

SUPPLEMENTARY FILE CONTENTS

Supplementary Table 1. Search strategy for electronic databases*..2

Supplementary Figure 1. The constructs of the Capability, Opportunity and Motivation Model of Behaviour Change (COM-B) that were integrated into the Health Belief Model (HBM) to synthesise the findings...4

Supplementary Table 2. Quality assessment of included qualitative studies (n=39).............5

Supplementary Table 3. Quality assessment of included quantitative studies (n=38). 8

Supplementary Table 4. Quality assessment of included mixed methods studies (n=7).....11

Supplementary Table 5. Interventions strategies for optimising risk perception and management of cardiometabolic pregnancy complications and future cardiometabolic disease amongst high-risk pregnant and postpartum women. 12

Supplementary Table 1. Search strategy for electronic databases*.

**DATABASE**	**Ovid MEDLINE***						
**STRATEGY**	**#1 AND #2 AND #3**						
**Concepts**	**Risk perception (pregnant/postpartum women)**				**Risk communication (health professionals)**		
**#1**	**Key words:** risk perception* OR risk appraisal* OR perceived risk* OR risk judgment* OR perceived susceptibilit* OR perceived vulnerabilit* OR perceived likelihood OR perceived threat* OR perceived severity OR susceptibility estimate* OR risk estimate* OR health risk assessment* OR risk awareness OR risk assessment* OR self-rated risk* OR risk viewpoint* OR health risk appraisal* OR health risk perception OR health attitude* OR "opinion of risk*" OR risk belief* **Subject headings:** Risk Assessment/			**OR**	**Key words:** risk communication OR risk messag* OR "communication of risk*" OR health risk communication **Subject headings:** Health Communication/		
**Concepts**	**GDM**		**HDP**		**IUGR/FGR**		**SPTB/PTB**
**#2**	**Key words:** gestational diabetes OR GDM OR pregnancy-induced diabetes **Subject headings:** Diabetes, Gestational/	**OR**	**Key words:** **"**hypertensive disorder* of pregnancy" OR "hypertension in pregnancy" OR preeclampsia OR eclampsia OR HDP OR pregnancy-induced hypertension OR	**OR**	**Key words:** IUGR OR intrauterine growth restriction OR low birth weight OR SGA OR "small for gestational age" OR fetal growth restriction OR FGR OR intrauterine growth retardation OR low birth-weight infant* OR very low birth weight infant*	**OR**	**Key words:** preterm birth* OR SPTB OR PTB OR spontaneous preterm birth* OR premature birth* OR premature infant* OR neonatal prematur* OR preterm infant* OR preterm deliver* OR premature deliver* OR "premature rupture of membranes" OR "preterm rupture

			gestational hypertension OR “high blood pressure in pregnancy” **Subject headings:** Hypertension, Pregnancy-Induced/		**Subject headings:** Fetal Growth Retardation/ OR Infant, Low Birth Weight/ OR Infant, Very Low Birth Weight/ OR Infant, Extremely Low Birth Weight/ OR Infant, Small for Gestational Age/		of membranes” OR premature labor OR preterm labor OR PROM **Subject headings:** Premature Birth/ OR Infant, Premature/ OR Labor Premature/ OR Obstetric Labor, Premature/ OR Infant, Extremely Premature/
**Concepts**	**Women**		**Mothers**		**Health professionals**		
**#3**	**Key words:** wom#n OR female OR womens health	**OR**	**Key words:** mother* OR m#m OR pregnan* OR postpartum period OR postnatal care	**OR**	**Key words:** health professional* OR allied health OR medical OR healthcare professional* OR healthcare provider* OR doctor* OR nurs* OR specialist* OR dieti#ian* OR dietetics OR clinician* OR practitioner* OR obstetrician* OR gynaecolog* OR endocrinolog* OR exercise physiolog* OR psycholog* OR physiotherap* OR midwif* OR family physician **Subject headings:** Allied Health Personnel/ OR Physicians/ OR Health Personnel/		

*Search words were modified as required for the other databases; CINAHL plus, Cochrane Library and APA PsychInfo.

Supplementary Figure 1. The constructs of the Capability, Opportunity and Motivation Model of Behaviour Change (COM-B) that were integrated into the Health Belief Model (HBM) to synthesise the findings.

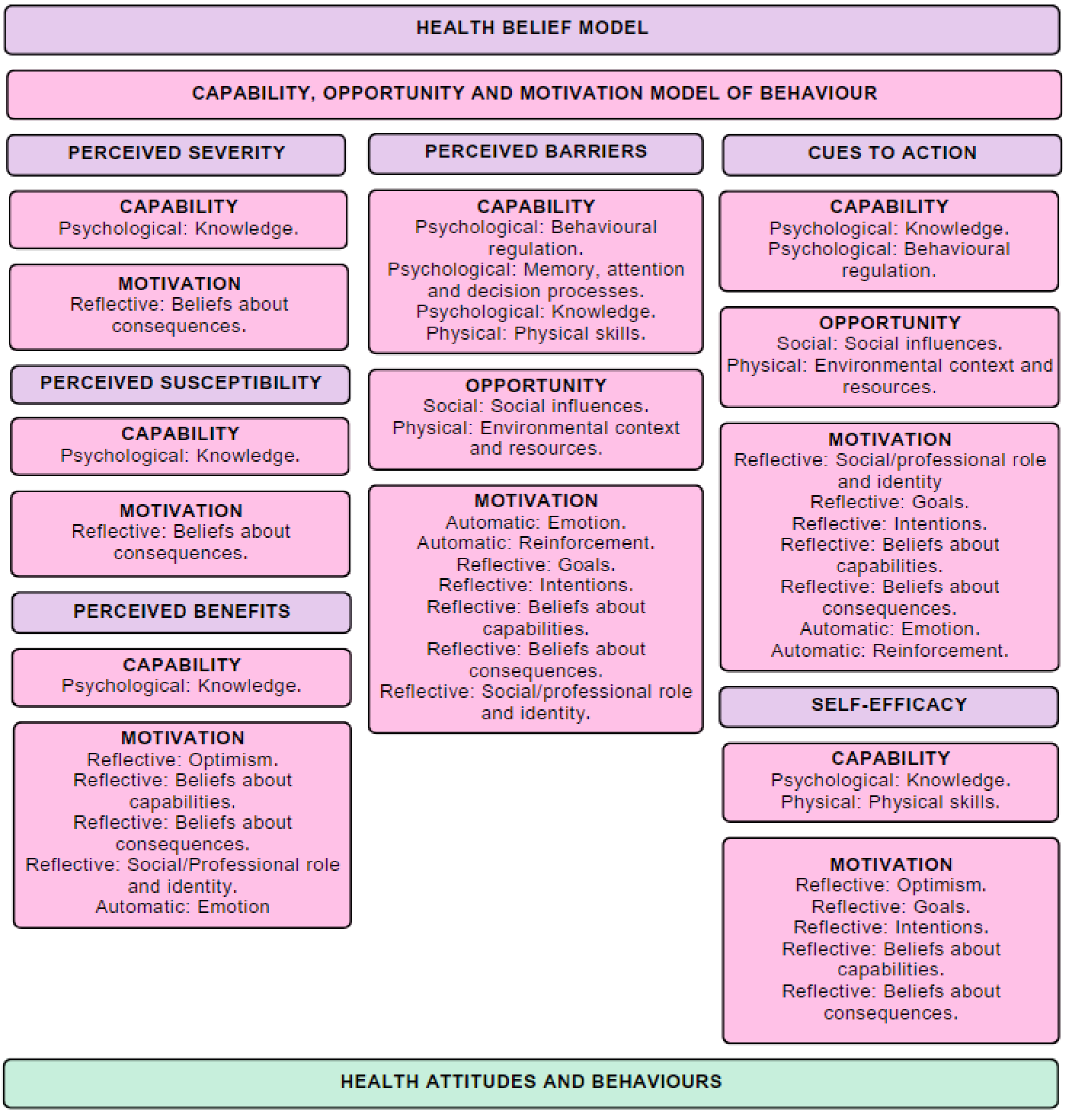

Purple represents the HBM. Pink represents the COM-B. Green represents influenced health attitudes and behaviours.

Supplementary Table 2. Quality assessment of included qualitative studies (n=39).

Author, Year	Study Design	Quality Assessment										
		Clear statement of aims	Appropriate to use qualitative methodology	Appropriate research design to meet aims	Appropriate recruitment strategy for aims	Collection of data addresses research issue	Adequate consideration of relationship between researcher(s) and participant(s)	Consideration of ethical issues	Rigorous data analysis	Clear statement of findings	Research value	**Overall grade (Low/ Moderate/ High)**
Abraham et al., 2014	Qualitative	Yes	Yes	Yes	Yes	Yes	No	Yes	Yes	Yes	Yes	High
Adu-Bonsaffoh et al., 2023	Qualitative	Yes	Yes	Yes	Yes	Yes	No	yes	yes	yes	yes	High
Bagger et al., 2021	Qualitative	Yes	Yes	Yes	Yes	Yes	No	Yes	Yes	Yes	Yes	High
Bennett et al., 2011	Qualitative	Yes	Yes	Yes	Yes	Yes	No	Yes	Yes	Yes	Yes	High
Bogale et al., 2020	Qualitative	Yes	Yes	Yes	Can't tell	Can't tell	No	Can't tell	Can't tell	Yes	Yes	Low
Brown et al., 2013	Qualitative	Yes	Yes	Yes	Yes	Yes	No	Yes	Yes	Yes	Yes	High
Collier et al., 2011	Qualitative	Yes	Yes	Yes	Yes	Yes	No	No	Yes	Yes	Yes	Moderate
Dennison et al., 2022	Qualitative	Yes	Yes	Yes	Yes	Yes	No	Yes	Yes	Yes	Yes	High
Dijkhuis et al., 2020	Qualitative	Yes	Yes	Yes	Yes	Yes	Yes	Yes	Yes	Yes	Yes	High
Eades et al., 2020	Qualitative	Yes	Yes	Yes	Yes	Yes	Yes	Yes	Yes	Yes	Yes	High
Ge et al., 2016	Qualitative	Yes	Yes	Yes	Yes	Yes	Yes	Yes	Yes	Yes	Yes	High
Ge et al., 2016	Qualitative	Yes	Yes	Yes	Yes	Yes	No	Yes	Yes	Yes	Yes	High

Graco et al., 2009	Qualitative	Yes	Yes	Yes	Yes	Yes	No	Yes	Yes	Yes	Yes	High
Gunn et al., 2020	Qualitative	Yes	Yes	Yes	Yes	Yes	No	Yes	Yes	Yes	Yes	High
Hirst et al., 2012	Qualitative	Yes	Yes	Yes	Yes	Yes	Yes	Yes	Yes	Yes	Yes	High
Hjelm et al., 2005	Qualitative	Yes	Yes	Yes	Yes	Yes	Yes	Yes	Yes	Yes	Yes	High
Hjelm et al., 2009	Qualitative	Yes	Yes	Yes	Yes	Yes	Yes	Yes	Yes	Yes	Yes	High
Hjelm et al., 2012	Qualitative	Yes	Yes	Yes	Yes	Yes	No	Yes	Yes	Yes	Yes	High
Hjelm et al., 2021	Qualitative	Yes	Yes	Yes	Can't tell	Yes	No	Yes	Yes	Yes	Yes	Moderate
Hjelm et al., 2022	Qualitative	Yes	Yes	Yes	Yes	Yes	No	Yes	Yes	Yes	Yes	High
Krompa et al., 2020	Qualitative	Yes	Yes	Yes	Yes	Yes	Yes	Yes	Yes	Yes	Yes	High
Lucas et al., 2022	Qualitative	Yes	Yes	Yes	Yes	Yes	No	Can't tell	Yes	Yes	Yes	Moderate
Nedergaard et al., 2023	Qualitative	Yes	Yes	Yes	Yes	Yes	No	Yes	Yes	Yes	Yes	High
Nielsen et al., 2022	Qualitative	Yes	Yes	Yes	Yes	Yes	Yes	Yes	Yes	Yes	Yes	High
Offomiyor et al., 2023	Qualitative	No	Yes	Yes	Yes	Yes	No	Yes	Yes	Yes	Yes	Moderate
Parsons et al., 2019	Qualitative	Yes	Yes	Yes	Yes	Yes	No	Yes	Yes	Yes	Yes	High
Poth et al., 2013	Qualitative	Yes	Yes	Yes	Yes	Yes	No	Yes	Yes	Yes	Yes	High
Qian et al., 2022	Qualitative	Yes	Yes	Yes	Yes	Yes	No	yes	Yes	yes	yes	High
Razee et al., 2010	Qualitative	Yes	Yes	Yes	Yes	Yes	No	Yes	Yes	Yes	Yes	High
Rossiter et al. 2022	Qualitative	Yes	Yes	Yes	Yes	Yes	No	Yes	Yes	Yes	Yes	High
Seely et al., 2013	Qualitative	Yes	Yes	Yes	Yes	Yes	No	Yes	Yes	Yes	Yes	High
Shang et al., 2021	Qualitative	Yes	Yes	Yes	Yes	Yes	No	Yes	Yes	Yes	Yes	High

Sharma et al., 2019	Qualitative	Yes	Yes	Yes	Yes	Yes	Yes	Yes	Yes	Yes	Yes	High
Skurnik et al., 2016	Qualitative	Yes	Yes	Yes	Yes	Yes	No	Yes	Yes	Yes	Yes	High
Stotz et al., 2019	Qualitative	Yes	Yes	Yes	Yes	Yes	No	Yes	Yes	Yes	Yes	High
Sunny et al., 2020	Qualitative	Yes	Yes	Yes	Yes	Yes	No	Yes	Yes	Yes	Yes	High
Tang et al., 2015	Qualitative	Yes	Yes	Yes	Yes	Yes	No	Yes	Yes	Yes	Yes	High
Teh et al., 2021	Qualitative	Yes	Yes	Yes	Yes	Yes	Yes	Yes	Yes	Yes	Yes	High
Toft et al., 2022	Qualitative	Yes	Yes	Yes	Yes	Yes	No	Yes	Yes	Yes	Yes	High

Supplementary Table 3. Quality assessment of included quantitative studies (n=38).

Author, Year	Study Design	Qualitative Assessment											
		Addresses a clearly focused research question	Study design is appropriate for answering the research question	Clearly described methods for selection of participants	The way the sample was obtained introduces bias	Participants represent the population the findings are referred to	Sample sized based on pre-study considerations of statistical power	Satisfactory response rate	Valid and reliable questionnaire(s)	Assessment of statistical significance	Confidence intervals for main results	Possible confounding factors not accounted for	Ability to apply results	Quality Assessment (Low/ Moderate/ High)
Akinwaare et al., 2020	Quantitative	Yes	Yes	Yes	Yes	No	Yes	Yes	Can't tell	Yes	No	No	Yes	Moderate
Aldridge et al., 2023	Quantitative	Yes	Yes	Yes	Yes	Yes	No	Yes	Yes	No	No	Yes	Yes	Low
Atkinson et al., 2023	Quantitative	Yes	Yes	Yes	Yes	Yes	No	No	Yes	Yes	No	Yes	Yes	Low
Bayrampour et al., 2012	Quantitative	Yes	Yes	Yes	Yes	No	Can't tell	Yes	Yes	Yes	No	No	Yes	Moderate
Beussink-Nelson et al., 2022	Quantitative	Yes	Yes	Yes	Yes	No	No	No	Yes	Yes	Yes	Yes	Yes	Low
Burgess et al., 2019	Quantitative	Yes	Yes	Yes	Yes	No	No	Yes	Yes	No	No	Yes	Yes	Low
Chuang et al., 2008	Quantitative	Yes	Yes	Yes	Yes	Yes	Can't tell	Yes	Can't tell	Yes	Yes	No	Yes	Moderate
Dobbs et al., 2021	Quantitative	Yes	Yes	Yes	Yes	No	No	Yes	Can't tell	Yes	No	Yes	Yes	Low
Feig et al., 1998	Quantitative	Yes	Yes	Yes	Yes	No	Can't tell	Yes	Yes	Yes	No	Yes	Yes	Low
Ferranti et al., 2014	Quantitative	Yes	Yes	Yes	Yes	No	No	Yes	Yes	Yes	No	Yes	Yes	Low
Goldstein et al., 2015	Quantitative	Yes	Yes	Yes	Yes	No	No	No	Can't tell	No	No	Yes	Yes	Low
Gray et al., 2020	Quantitative	Yes	Yes	Yes	Yes	No	Yes	Yes	Yes	Yes	Yes	Yes	Yes	Moderate
Gray et al., 2021	Quantitative	Yes	Yes	Yes	Yes	No	No	Yes	Yes	Yes	No	Yes	Yes	Low

Harrison et al., 2012	Quantitative	Yes	Yes	Yes	Yes	Yes	Yes	Yes	Yes	Yes	Yes	No	Yes	High
Huang et al., 2022	Quantitative	Yes	Yes	Yes	Yes	Yes	Yes	Can’t tell	Yes	Yes	No	Yes	yes	Moderate
Hutchesson et al., 2018	Quantitative	Yes	Yes	Yes	Yes	No	No	Yes	Can't tell	No	No	Yes	Yes	Low
Joshi et al., 2020	Quantitative	Yes	Yes	Yes	Yes	Yes	No	Yes	Yes	Yes	No	No	Yes	Moderate
Kaiser et al., 2016	Quantitative	Yes	Yes	Yes	Yes	Yes	No	Yes	Can't tell	Yes	Yes	Yes	Yes	Moderate
Kim et al., 2007	Quantitative	Yes	Yes	Yes	No	No	No	Yes	Can't tell	Yes	No	No	Yes	Low
Kim et al., 2020	Quantitative	Yes	Yes	Yes	Yes	No	Yes	Yes	Yes	Yes	Yes	Yes	Yes	Moderate
Malcolm et al., 2009	Quantitative	Yes	Yes	Yes	Yes	No	No	No	Can’t tell	Yes	Yes	Yes	Yes	Low
Mekie et al., 2021	Quantitative	Yes	Yes	Yes	No	Yes	Yes	Yes	Can't tell	Yes	Yes	Yes	Yes	Moderate
Minsart et al., 2014	Quantitative	Yes	Yes	Yes	Yes	Yes	No	Yes	Can't tell	Yes	Yes	No	Yes	Moderate
Morrison et al., 2010	Quantitative	Yes	Yes	Yes	Yes	No	No	No	Yes	Yes	Yes	No	Yes	Low
Mukerji et al., 2016	Quantitative	Yes	Yes	Yes	No	No	No	No	Yes	Yes	Yes	Yes	Yes	Moderate
Nitert et al., 2011	Quantitative	Yes	Yes	Yes	No	No	Yes	Yes	Can't tell	Yes	Yes	Yes	Yes	Moderate
Okely et al., 2019	Quantitative	Yes	Yes	Yes	Yes	No	No	No	No	Yes	No	No	Yes	Low
O’Reilly et al., 2021	Quantitative	Yes	Yes	Yes	No	No	No	Yes	Yes	Yes	Yes	Yes	Yes	Moderate
Park et al., 2018	Quantitative	Yes	Yes	Yes	Yes	No	Yes	Yes	Yes	Yes	Yes	No	Yes	Moderate
Passey et al., 2012	Quantitative	Yes	Yes	Yes	No	Yes	Yes	Yes	Yes	Yes	Yes	No	Yes	High
Qian et al., 2023	Quantitative	Yes	Yes	Yes	No	No	No	No	Yes	Yes	No	Yes	Yes	Low
Roth et al., 2020	Quantitative	Yes	Yes	Yes	Yes	No	No	Can’t tell	Yes	Yes	No	Yes	Yes	Low
Stacy et al., 1994	Quantitative	Yes	Yes	Yes	Yes	No	No	Yes	Can't tell	Yes	No	Yes	Yes	Low
Sutherland et al., 2020	Quantitative	Yes	Yes	Yes	Yes	No	No	Yes	Yes	Yes	No	Yes	Yes	Low

Swan et al., 2007	Quantitative	Yes	Yes	Yes	No	Yes	No	Yes	Yes	Yes	No	No	Yes	Moderate
Traylor et al., 2016	Quantitative	Yes	Yes	Yes	Yes	No	No	Yes	Yes	Yes	No	Yes	Yes	Low
Vu et al., 2022	Quantitative	Yes	Yes	Yes	No	Yes	No	Can't tell	Yes	Yes	Yes	No	Yes	Moderate
Zera et al., 2013	Quantitative	Yes	Yes	Yes	Yes	No	No	Yes	Yes	Yes	No	Yes	Yes	Low

Supplementary Table 4. Quality assessment of included mixed methods studies (n=7).

**Author, Year**	**Study Design**	**Quality Assessment**							
		Clear research question(s)	Collected data enables addressing of research question(s)	Adequate rationale for using mixed-methods design	Different components of the study effectively integrated to answer research question(s)	Outputs of the integration of qualitative and quantitative results adequately addressed	Divergences and inconsistencies between qualitative and quantitative results adequately addressed	Different components of the study adhere to the quality criteria of each tradition of the methods involved	**Quality Assessment (Low/ Moderate/ High)**
Brown et al., 2017	Mixed methods	Yes	Yes	Yes	Yes	Yes	Yes	Yes	High
Jelsma et al., 2016	Mixed methods	Yes	Yes	Yes	Yes	Yes	Yes	Yes	High
Jones et al., 2012	Mixed methods	Yes	Yes	Yes	Yes	Yes	Yes	Yes	High
Jones et al., 2015	Mixed methods	Yes	Yes	Yes	Yes	Yes	Yes	Yes	High
Nicklas et al., 2011	Mixed methods	Yes	Yes	Yes	Yes	Yes	Yes	Yes	High
Singh et al., 2018	Mixed methods	Yes	Yes	Yes	Yes	Yes	Yes	Yes	High
Sterne et al., 2011	Qualitative	Yes	Yes	Yes	Yes	Yes	Yes	Yes	High

Supplementary Table 5. Interventions strategies for optimising risk perception and management of cardiometabolic pregnancy complications and future cardiometabolic disease amongst high-risk pregnant and postpartum women.

Health Belief Model (HBM) constructs and associated key findings	Capability, Opportunity and Motivation Model of Behaviour Change (COM-B) constructs and relevant Theoretical Domains Framework (TDF) domains	Intervention functions to promote behaviour change	Behaviour Change Techniques (BCTs) related to Intervention Functions (BCT number)	Example proposed intervention strategies to optimise cardiometabolic risk perception and management in high-risk pregnant and postpartum women
**Perceived susceptibility:**<br>• Low-moderate knowledge of cardiometabolic pregnancy complications as a risk factor for future cardiometabolic disease.<br>• Low-moderate risk perception for cardiometabolic pregnancy complications and future cardiometabolic disease.	***CAPABILITY - Psychological***			
	**Knowledge**	**Education**	Information about health consequences (BCT 5.1)	Provide high-risk women with education/information (written, verbal and/or visual) about general and personal risk magnitude, risk factors and health consequences (e.g., association between lifestyle behaviours and cardiometabolic pregnancy complication development, pregnancy specific maternal and foetal health consequences of cardiometabolic pregnancy complications, postpartum specific maternal health consequences of cardiometabolic pregnancy complications). Additionally, provide education/information on health condition severity as well as why, when and how to engage in postpartum cardiometabolic disease risk screening. To be spoken in an empathetic and understanding way.
			Credible source (BCT 9.1)	Accredited healthcare professional to provide education (e.g. general practitioner, obstetrician, nurse, midwife, accredited practicing dietitian)
			Information about social and environmental consequences (BCT 5.3)	Women to be provided with information on risks, implications and the benefits of reducing their risk for their families, for example, explaining that how women can be a positive role model and improve the health of their children may promote engagement with risk information and risk reduction strategies, such as screening and healthy lifestyle behaviours.
			Prompts/cues (BCT 7.1)	Education/information provided about general and personal risk magnitude, risk factors and health consequences and health condition severity can be used as a cue to action for women to engage in risk reducing lifestyle behaviours before, during and after pregnancy.
	MOTIVATION - Reflective			
	**Beliefs about consequences**	**Education, Persuasion, Modelling**	Information about health consequences (BCT 5.1)	As per capability, psychological capability, knowledge (perceived susceptibility).

			Credible source<br>(BCT 9.1)	As per capability, psychological capability, knowledge (perceived susceptibility).
			Information about social and environmental consequences<br>(BCT 5.3)	As per capability, psychological capability, knowledge (perceived susceptibility).
			Prompts/cues<br>(BCT 7.1)	As per capability, psychological capability, knowledge (perceived susceptibility).
**Perceived severity:**<br><br>• Lack of clarity regarding the severity of cardiometabolic pregnancy complications and consequences***.***	***CAPABILITY - Psychological***			
	**Knowledge**	**Education**	Information about health consequences<br>(BCT 5.1)	As per capability, psychological capability, knowledge (perceived susceptibility).
			Credible source<br>(BCT 9.1)	As per capability, psychological capability, knowledge (perceived susceptibility).
			Information about social and environmental consequences<br>(BCT 5.3)	As per capability, psychological capability, knowledge (perceived susceptibility).
			Prompts/cues<br>(BCT 7.1)	As per capability, psychological capability, knowledge (perceived susceptibility).
	MOTIVATION - Reflective			
	**Beliefs about consequences**	**Education, Persuasion, Modelling**	Information about health consequences<br>(BCT 5.1)	As per capability, psychological capability, knowledge (perceived susceptibility).
			Credible source<br>(BCT 9.1)	As per capability, psychological capability, knowledge (perceived susceptibility).
			Information about social and environmental consequences<br>(BCT 5.3)	As per capability, psychological capability, knowledge (perceived susceptibility).
			Prompts/cues<br>(BCT 7.1)	As per capability, psychological capability, knowledge (perceived susceptibility).
**Perceived barriers:**<br><br>• Lack of social/ healthcare professional	***CAPABILITY – Psychological***			
	**Knowledge**	**Education**	Information about health consequences<br>(BCT 5.1)	As per capability, psychological capability, knowledge (perceived susceptibility).<br><br>Additionally, provide high-risk women with education/information (written, verbal and/or visual) on benefits of engaging in a healthy lifestyle (nutrition, physical activity, mental and emotional wellbeing) and regular cardiometabolic screening

support, physical/mental/emotional stress, fatigue, burden, low risk perception, low perceived severity, low knowledge, lack of resources/skills, finances, time, prioritisation of baby's health.				in order to reduce their risk of cardiometabolic pregnancy complications and future cardiometabolic disease. To be provided in an empowering and motivating way.
			Credible source (BCT 9.1)	As per capability, psychological capability, knowledge (perceived susceptibility).
			Prompts/cues (BCT 7.1)	Encourage women to introduce or define environmental or social stimulus for the purpose of prompting or cueing positive self-talk and healthful behaviours (e.g. smart watch reminder to walk every 30 minutes, a positive affirmation written in dairy or on a post it notes stuck on the bathroom mirror, placing water bottle on desk as a reminder to drink water, a daily calendar reminder to eat two pieces of fruit). Assist women to identify internal motivators for behaviour change. Encourage them to utilise these as a prompt/cue to continue with healthy behaviour change.
	**Behavioural regulation**	**Education, Enablement, Modelling, Training**	Action planning (BCT 1.4)	Assist women in creating a plan on how to achieve their SMART goals set. Prompt planning the performance of a particular health behaviour (e.g. running, walking) at a particular time (e.g. before the kids wake up, when alarm goes off at 6:00am, during lunch break) on certain days of the week (e.g. Monday, Wednesday, Friday).
			Adding objects to the environment (BCT 12.5)	Encourage women to add objects to their environment in order to facilitate behavioural change (e.g. ensuring there is always fresh fruit at home and the pantry contains healthy snacks, buying hand held weights to facilitate muscle strengthening home workouts, buying a yoga mat to facilitate yoga and meditation, print out visuals of how to perform pelvic floor exercises and utilise them whilst exercising, purchasing a stand-up desk for the at home office to reduce sitting time).
			Behavioural practice/ rehearsal (BCT 8.1)	Encourage women to practice and continue healthy behaviours, even if several attempts are required (e.g. practice cooking healthy meals and preparing healthy snacks, practice mobility exercises and pelvic floor strengthening exercises to increase mobility and strength pelvic floor for gradual return to running postpartum).
			Demonstration of the behaviour (BCT 6.1)	Provide visual, audio and audio-visual information regarding preparing healthy meals and snacks, exercising safely during pregnancy and postpartum and engaging in other health related behaviours that help reduce cardiometabolic disease risk (e.g. cooking videos, workout videos, step-by-step guided meditation).
			Habit formation (BCT 8.3)	Prompt rehearsal and repetition of behaviours that overcome certain barriers to engaging in healthful behaviours (e.g. packed lunches and snacks for work he night before to avoid lack of time in morning that leads to buying less healthy foods outside the home, get workout gear ready and place it out the night before,

				utilise supermarket sale catalogue when writing shopping less to save money on nutritious foods, perform exercise snacks throughout the day during busy days with low time, meditate every morning for 2 minutes before getting out of bed to help overcome stress).
			Information about health consequences (BCT 5.1)	As per capability, psychological capability, knowledge (perceived barriers).
			Credible source (BCT 9.1)	As per capability, psychological capability, knowledge (perceived susceptibility).
			Instruction on how to perform the behaviour (BCT 4.1)	Instruct women on how to eat healthy, prepare meals and snacks and exercise in a manner conducive to reducing cardiometabolic disease risk during and after pregnancy (e.g. provide instructions on how to create a healthy balanced plate, provide instructions on how to perform pelvic floor strengthening exercises postpartum, provide instructions on how to read food labels in order to choose packaged items that are high in fibre and low in fat, salt and sugar).
			Prompts/cues (BCT 7.1)	As per capability, psychological capability, knowledge (perceived barriers).
			Problem solving (BCT 1.2)	Encourage women to identify potential barriers and facilitators to lifestyle change and then possible ways to overcome barriers (e.g. if it is raining walk on the treadmill or carry an umbrella, lay out gym gear the night before ready to go for morning, pre-prepare lunch and snacks the night before for work the next day to avoid buying take-away due to lack of time in the morning to prepare lunch, set alarm 5minutes earlier to meditate before getting out of bed, join a group class if lacking motivation to exercise alone, break physical activity into small chunks scattered throughout the day).
			Reduce negative emotions (BCT 11.2)	Advise women on the use of stress management skills (e.g. meditation, deep breathing, journaling, getting adequate sleep) to help reduce mental and emotional stress, which may be impacting women's ability to implement and maintain healthy lifestyle behaviours or engage with screening.
			Restructuring the physical environment (BCT 12.1)	Help women to identify changes in their physical environment that can be made to assist with achieving their behavioural SMART goal(s) to reduce cardiometabolic risk (other than prompts/cues, rewards/punishments) (e.g. placing less nutritious snacks in a cupboard or drawer that is inconvenient to get to, purchasing healthy snacks and placing them at the front of the fridge or pantry, placing a bowl of fresh fruit on the kitchen bench, placing a plate of cut up fresh fruit on the kitchen bench when at home to snack on throughout the day, clearing/making a space in the house to do exercise at home).

			Restructuring the social environment (BCT 12.2)	Advise women to minimise time spent with individuals in their social environment who engage in behaviours that contraindicate living a healthy lifestyle, and increase time spent with those engaging in behaviours that coincide with living a healthy lifestyle (e.g. joining a social walking group, arranging to meet with friends in a park as opposed to a restaurant).
			Social support (unspecified) (BCT 3.1)	Arrange for social support(s) (e.g. partner, close family member, close friend) to provide verbal encouragement, positive words of affirmation, emotional and moral support regarding initiation and continuation of health lifestyle behaviour change.
			Social support (emotional) (BCT 3.3)	
			Social support (practical) (BCT 3.2)	Encourage women to speak to their social support(s) (e.g. partner, close family member, close friend) about their lifestyle related SMART goal(s) and how and when their social support(s) can assist them in making healthy lifestyle changes (e.g. buying healthy snacks, cooking healthy meals). Encourage women to encourage their social support(s) network to also engage in healthy lifestyle behaviours (e.g. also eat healthy and exercise regularly).<br><br>Refer women to appropriate community groups (e.g. pregnancy and postpartum groups, group exercise classes, community cooking lessons) if women need additional support with engaging in and maintain healthy lifestyle changes.
	**Memory, attention and decision processes**	**Enablement, Environmental restructuring, Training**	Action planning (BCT 1.4)	As per capability, psychological capability, behavioural regulation (perceived barriers).
			Adding objects to the environment (BCT 12.5)	As per capability, psychological capability, behavioural regulation (perceived barriers).
			Behavioural practice/ rehearsal (BCT 8.1)	As per capability, psychological capability, behavioural regulation (perceived barriers).
			Demonstration of the behaviour (BCT 6.1)	As per capability, psychological capability, behavioural regulation (perceived barriers).
			Instruction on how to perform the behaviour (BCT 4.1)	As per capability, psychological capability, behavioural regulation (perceived barriers).
			Prompts/cues (BCT 7.1)	As per capability, psychological capability, knowledge (perceived barriers).

			Habit formation (BCT 8.3)	As per capability, psychological capability, behavioural regulation (perceived barriers).
			Problem solving (BCT 1.2)	As per capability, psychological capability, behavioural regulation (perceived barriers).
			Reduce negative emotions (BCT 11.2)	As per capability, psychological capability, behavioural regulation (perceived barriers).
			Restructuring the social environment (BCT 12.2)	As per capability, psychological capability, behavioural regulation (perceived barriers).
			Social support (unspecified) (BCT 3.1)	As per capability, psychological capability, behavioural regulation (perceived barriers).
			Social support (emotional) (BCT 3.3)	
			Social support (practical) (BCT 3.2)	As per capability, psychological capability, behavioural regulation (perceived barriers).
	CAPABILITY – Physical			
	**Physical skills**	**Training**	Behavioural practice/ rehearsal (BCT 8.1)	As per capability, psychological capability, behavioural regulation (perceived barriers).
			Demonstration of the behaviour (BCT 6.1)	As per capability, psychological capability, behavioural regulation (perceived barriers).
			Instruction on how to perform the behaviour (BCT 4.1)	As per capability, psychological capability, behavioural regulation (perceived barriers).
			Habit formation (BCT 8.3)	As per capability, psychological capability, behavioural regulation (perceived barriers).
	MOTIVATION – Reflective			
	**Goals**	**Education, Coercion, Incentivisation, Modelling, Persuasion**	Action planning (BCT 1.4)	As per capability, psychological capability, behavioural regulation (perceived barriers).
			Adding objects to the environment (BCT 12.5)	As per capability, psychological capability, behavioural regulation (perceived barriers).

			Demonstration of the behaviour (BCT 6.1)	As per capability, psychological capability, behavioural regulation (perceived barriers).
			Information about health consequences (BCT 5.1)	As per capability, psychological capability, knowledge (perceived barriers).
			Credible source (BCT 9.1)	
			Problem solving (BCT 1.2)	As per capability, psychological capability, behavioural regulation (perceived barriers).
			Prompts/cues (BCT 7.1)	As per capability, psychological capability, knowledge (perceived barriers).
			Restructuring the physical environment (BCT 12.1)	As per capability, psychological capability, behavioural regulation (perceived barriers).
			Social support (unspecified) (BCT 3.1)	As per capability, psychological capability, behavioural regulation (perceived barriers).
			Social support (emotional) (BCT 3.3)	
			Social support (practical) (BCT 3.2)	As per capability, psychological capability, behavioural regulation (perceived barriers).
	**Intentions**	**Education, Coercion, Incentivisation, Modelling, Persuasion**	Demonstration of the behaviour (BCT 6.1)	As per capability, psychological capability, behavioural regulation (perceived barriers).
			Information about health consequences (BCT 5.1)	As per capability, psychological capability, knowledge (perceived barriers).
			Credible source (BCT 9.1)	As per capability, psychological capability, knowledge (perceived susceptibility).
			Prompts/cues (BCT 7.1)	As per capability, psychological capability, knowledge (perceived barriers).
	**Beliefs about capabilities**	**Education, Enablement, Modelling, Persuasion**	Action planning (BCT 1.4)	As per capability, psychological capability, behavioural regulation (perceived barriers).
			Adding objects to the environment	As per capability, psychological capability, behavioural regulation (perceived barriers).

			(BCT 12.5)	
			Demonstration of the behaviour (BCT 6.1)	As per capability, psychological capability, behavioural regulation (perceived barriers).
			Information about health consequences (BCT 5.1)	As per capability, psychological capability, knowledge (perceived barriers).
			Credible source (BCT 9.1)	As per capability, psychological capability, knowledge (perceived susceptibility).
			Problem solving (BCT 1.2)	As per capability, psychological capability, behavioural regulation (perceived barriers).
			Prompts/cues (BCT 7.1)	As per capability, psychological capability, knowledge (perceived barriers).
			Reduce negative emotions (BCT 11.2)	As per capability, psychological capability, behavioural regulation (perceived barriers).
			Social support (unspecified) (BCT 3.1)	As per capability, psychological capability, behavioural regulation (perceived barriers).
			Social support (emotional) (BCT 3.3)	
			Social support (practical) (BCT 3.2)	As per capability, psychological capability, behavioural regulation (perceived barriers).
			Restructuring the physical environment (BCT 12.1)	As per capability, psychological capability, behavioural regulation (perceived barriers).
	**Beliefs about consequences**	**Education, Persuasion, Modelling**	Demonstration of the behaviour (BCT 6.1)	As per capability, psychological capability, behavioural regulation (perceived barriers).
			Information about health consequences (BCT 5.1)	As per capability, psychological capability, knowledge (perceived barriers).
			Credible source (BCT 9.1)	As per capability, psychological capability, knowledge (perceived susceptibility).
			Prompts/cues (BCT 7.1)	As per capability, psychological capability, knowledge (perceived barriers).

	**Social/professional role and identity**	**Education, Modelling, Persuasion**	Demonstration of the behaviour (BCT 6.1)	As per capability, psychological capability, behavioural regulation (perceived barriers).
			Information about health consequences (BCT 5.1)	As per capability, psychological capability, knowledge (perceived barriers).
			Credible source (BCT 9.1)	As per capability, psychological capability, knowledge (perceived susceptibility).
			Prompts/cues (BCT 7.1)	As per capability, psychological capability, knowledge (perceived barriers).
	MOTIVATION – Automatic			
	**Emotion**	**Coercion, Enablement Incentivisation, Modelling, Persuasion**	Adding objects to the environment (BCT 12.5)	As per capability, psychological capability, behavioural regulation (perceived barriers).
			Action planning (BCT 1.4)	As per capability, psychological capability, behavioural regulation (perceived barriers).
			Demonstration of the behaviour (BCT 6.1)	As per capability, psychological capability, behavioural regulation (perceived barriers).
			Information about health consequences (BCT 5.1)	As per capability, psychological capability, knowledge (perceived barriers).
			Credible source (BCT 9.1)	As per capability, psychological capability, knowledge (perceived susceptibility).
			Problem solving (BCT 1.2)	As per capability, psychological capability, behavioural regulation (perceived barriers).
			Restructuring the physical environment (BCT 12.1)	As per capability, psychological capability, behavioural regulation (perceived barriers).
			Social support (unspecified) (BCT 3.1)	As per capability, psychological capability, behavioural regulation (perceived barriers).
			Social support (emotional) (BCT 3.3)	
			Social support (practical) (BCT 3.2)	As per capability, psychological capability, behavioural regulation (perceived barriers).

			Reduce negative emotions (BCT 11.2)	As per capability, psychological capability, behavioural regulation (perceived barriers).
	**Reinforcement**	**Training, Incentivisation, Coercion, Environmental restructuring**	Adding objects to the environment (BCT 12.5)	As per capability, psychological capability, behavioural regulation (perceived barriers).
			Demonstration of the behaviour (BCT 6.1)	As per capability, psychological capability, behavioural regulation (perceived barriers).
			Instruction on how to perform the behaviour (BCT 4.1)	As per capability, psychological capability, behavioural regulation (perceived barriers).
			Prompts/cues (BCT 7.1)	As per capability, psychological capability, knowledge (perceived barriers).
			Restructuring the physical environment (BCT 12.1)	As per capability, psychological capability, behavioural regulation (perceived barriers).
			Social support (unspecified) (BCT 3.1)	As per capability, psychological capability, behavioural regulation (perceived barriers).
			Social support (emotional) (BCT 3.3)	
			Social support (practical) (BCT 3.2)	As per capability, psychological capability, behavioural regulation (perceived barriers).
	OPPORTUNITY – Physical			
	**Environmental Context and Resources**	**Enablement, Environmental restructuring, Restriction, Training**	Action planning (BCT 1.4)	As per capability, psychological capability, behavioural regulation (perceived barriers).
			Adding objects to the environment (BCT 12.5)	As per capability, psychological capability, behavioural regulation (perceived barriers).
			Behavioural practice/ rehearsal (BCT 8.1)	As per capability, psychological capability, behavioural regulation (perceived barriers).
			Demonstration of the behaviour	As per capability, psychological capability, behavioural regulation (perceived barriers).

			(BCT 6.1)	
			Instruction on how to perform the behaviour (BCT 4.1)	As per capability, psychological capability, behavioural regulation (perceived barriers).
			Problem solving (BCT 1.2)	As per capability, psychological capability, behavioural regulation (perceived barriers).
			Prompts/cues (BCT 7.1)	As per capability, psychological capability, knowledge (perceived barriers).
			Reduce negative emotions (BCT 11.2)	As per capability, psychological capability, behavioural regulation (perceived barriers).
			Restructuring the physical environment (BCT 12.1)	As per capability, psychological capability, behavioural regulation (perceived barriers).
			Social support (unspecified) (BCT 3.1)	As per capability, psychological capability, behavioural regulation (perceived barriers).
			Social support (emotional) (BCT 3.3)	
			Social support (practical) (BCT 3.2)	As per capability, psychological capability, behavioural regulation (perceived barriers).
	OPPORTUNITY – Social			
	**Social Influences**	**Enablement, Environmental restructuring, Modelling, Restriction**	Action planning (BCT 1.4)	As per capability, psychological capability, behavioural regulation (perceived barriers).
			Adding objects to the environment (BCT 12.5)	As per capability, psychological capability, behavioural regulation (perceived barriers).
			Demonstration of the behaviour (BCT 6.1)	As per capability, psychological capability, behavioural regulation (perceived barriers).
			Problem solving (BCT 1.2)	As per capability, psychological capability, behavioural regulation (perceived barriers).
			Prompts/cues (BCT 7.1)	As per capability, psychological capability, knowledge (perceived barriers).
			Reduce negative emotions	As per capability, psychological capability, behavioural regulation (perceived barriers).

			(BCT 11.2)	
			Social support (unspecified) (BCT 3.1)	As per capability, psychological capability, behavioural regulation (perceived barriers).
			Social support (emotional) (BCT 3.3)	
			Social support (practical) (BCT 3.2)	As per capability, psychological capability, behavioural regulation (perceived barriers).
			Restructuring the physical environment (BCT 12.1)	As per capability, psychological capability, behavioural regulation (perceived barriers).
**Self-efficacy:**<br><br>• Uncertain about personal ability to reduce risk.	***CAPABILITY – Psychological***			
	**Knowledge**	**Education**	Information about health consequences (BCT 5.1)	As per capability, psychological capability, knowledge (perceived barriers).
			Credible source (BCT 9.1)	As per capability, psychological capability, knowledge (perceived susceptibility).
	CAPBILITY – Physical			
	**Physical skills**	**Training**	Feedback on behaviour (BCT 2.2)	Review and provide positive/ practical feedback on optimising healthy lifestyle behaviours to increase women's confidence to engage those behaviours (e.g. compare intake of each food group to dietary guidelines and provide practical advice on how they can optimise their dietary intake).
			Feedback of outcome(s) of behaviour (BCT 2.7)	Provide feedback on the outcome of the performance of health behaviours to help demonstrate to women how their behaviour may be contributing to cardiometabolic risk profile (e.g. exploring how healthy lifestyle behaviours have resulted in improvements to medical/ blood test results or healthy weight change/loss during and after pregnancy).
			Self-talk (BCT 15.4)	Facilitate positive self-talk, either aloud and/or inside own head (e.g. 'I can reduce my risk', 'I can overcome barriers I am faced with', 'I am going to get healthier', 'I can do this', 'I am strong enough to do this') to increase women's confidence in reducing their cardiometabolic risk
			Verbal persuasion about capability (BCT 15.1)	Encourage and inform women that they can successfully reduce their cardiometabolic disease risk during and after pregnancy by engaging in a healthy lifestyle, arguing against self-doubts and assuring them that they can and will succeed (e.g. explaining that women who experienced a

				cardiometabolic pregnancy complication or have strong family history of cardiometabolic disease still have the opportunity to reduce future cardiometabolic disease risk and avoid experiencing a cardiometabolic pregnancy complication in subsequent pregnancies).
	MOTIVATION - Reflexive			
	**Optimism**	**Education, Enablement, Modelling, Persuasion**	Information about health consequences (BCT 5.1)	As per capability, psychological capability, knowledge (perceived barriers).
			Credible source (BCT 9.1)	As per capability, psychological capability, knowledge (perceived susceptibility).
			Feedback on behaviour (BCT 2.2)	As per capability, physical capability, physical skills (self-efficacy).
			Feedback of outcome(s) of behaviour (BCT 2.7)	As per capability, physical capability, physical skills (self-efficacy).
			Prompts/cues (BCT 7.1)	Encourage women to introduce or define environmental or social stimulus for the purpose of prompting or cueing positive self-talk and self-belief (e.g. a positive affirmation written in dairy or on a post it notes stuck on the bathroom mirror).
			Self-talk (BCT 15.4)	As per capability, physical capability, physical skills (self-efficacy).
			Reduce negative emotions (BCT 11.2)	Advise women on ways of reducing negative emotions, including self-doubt, stress and anxiety associated with being labelled as high risk for cardiometabolic disease development (e.g. use of positive self-talk, positive affirmations and stress management skills such as journaling, deep breathing, yoga and meditation).
			Social support (unspecified) (BCT 3.1)	As per capability, psychological capability, behavioural regulation (perceived barriers).
			Social support (emotional) (BCT 3.3)	
			Social comparison (BCT 6.2)	Provide written, verbal and/or visual examples of real women and/or data relating to the effect of lifestyle change in reducing cardiometabolic disease risk during and after a complicated pregnancy as an exemplar for women and to reinforce the idea that they can reduce their cardiometabolic disease risk during and after pregnancy by engaging in a healthy lifestyle (even if non-modifiable risk factors, such as family history, are present).

	**Goals**	**Education, Coercion, Enablement, Modelling, Persuasion, Incentivisation,**	Feedback on behaviour (BCT 2.2)	As per capability, physical capability, physical skills (self-efficacy).
			Feedback of outcome(s) of behaviour (BCT 2.7)	As per capability, physical capability, physical skills (self-efficacy).
			Information about health consequences (BCT 5.1)	As per capability, psychological capability, knowledge (perceived barriers).
			Credible source (BCT 9.1)	As per capability, psychological capability, knowledge (perceived susceptibility).
			Prompts/cues (BCT 7.1)	As per motivation, reflexive motivation, optimism (self-efficacy).
			Self-talk (BCT 15.4)	As per capability, physical capability, physical skills (self-efficacy).
			Reduce negative emotions (BCT 11.2)	Advise women on ways of reducing negative emotions, including self-doubt, stress and anxiety associated with being labelled as high risk for cardiometabolic disease development (e.g. use of positive self-talk, positive affirmations and stress management skills such as journaling, deep breathing, yoga and meditation) to improve women's confidence in engaging with healthy lifestyle behaviours.
			Social support (unspecified) (BCT 3.1)	As per capability, psychological capability, behavioural regulation (perceived barriers).
			Social support (emotional) (BCT 3.3)	
			Social comparison (BCT 6.2)	As per motivation, reflexive motivation, optimism (self-efficacy).
	**Intention**	**Education, Coercion, Incentivisation, Modelling, Persuasion**	Feedback on behaviour (BCT 2.2)	As per capability, physical capability, physical skills (self-efficacy).
			Feedback of outcome(s) of behaviour (BCT 2.7)	As per capability, physical capability, physical skills (self-efficacy).

			Information about health consequences (BCT 5.1)	As per capability, psychological capability, knowledge (perceived barriers).
			Credible source (BCT 9.1)	As per capability, psychological capability, knowledge (perceived susceptibility).
			Prompts/cues (BCT 7.1)	As per motivation, reflexive motivation, optimism (self-efficacy).
	**Beliefs about consequences**	**Education, Persuasion, Modelling**	Information about health consequences (BCT 5.1)	As per capability, psychological capability, knowledge (perceived barriers).
			Credible source (BCT 9.1)	As per capability, psychological capability, knowledge (perceived susceptibility).
			Feedback on behaviour (BCT 2.2)	As per capability, physical capability, physical skills (self-efficacy).
			Feedback of outcome(s) of behaviour (BCT 2.7)	As per capability, physical capability, physical skills (self-efficacy).
	**Beliefs about capabilities**	**Education, Enablement, Modelling, Persuasion**	Feedback on behaviour (BCT 2.2)	As per capability, physical capability, physical skills (self-efficacy).
			Feedback of outcome(s) of behaviour (BCT 2.7)	As per capability, physical capability, physical skills (self-efficacy).
			Information about health consequences (BCT 5.1)	As per capability, psychological capability, knowledge (perceived barriers).
			Credible source (BCT 9.1)	As per capability, psychological capability, knowledge (perceived susceptibility).
			Prompts/cues (BCT 7.1)	As per motivation, reflexive motivation, optimism (self-efficacy).
			Social support (unspecified) (BCT 3.1)	As per capability, psychological capability, behavioural regulation (perceived barriers).
			Social support (emotional) (BCT 3.3)	
			Social comparison	As per motivation, reflexive motivation, optimism (self-efficacy).

			(BCT 6.2)	
**Cues to action:**<br>• Emotional/social/healthcare professional support, family history, high risk perception, internal motivation, role modelling.	***CAPABILITY - Psychological***			
	**Knowledge**	**Education**	Information about health consequences (BCT 5.1)	As per capability, psychological capability, knowledge (perceived susceptibility).
			Credible source (BCT 9.1)	As per capability, psychological capability, knowledge (perceived susceptibility).
			Information about social and environmental consequences (BCT 5.3)	As per capability, psychological capability, knowledge (perceived susceptibility).
			Prompts/cues (BCT 7.1)	As per capability, psychological capability, knowledge (perceived susceptibility).
	OPPORTUNITY – Physical			
	**Environmental Context and Resources**	**Enablement, Environmental restructuring, Restriction, Training**	Adding objects to the environment (BCT 12.5)	Provide flyers and posters to be displayed in GP clinics, hospitals and community centres that educate women about risk factors for cardiometabolic pregnancy complications, their link to future cardiometabolic disease postpartum, the importance of screening and living a healthy lifestyle to reduce their risk.
			Prompts/cues (BCT 7.1)	As per capability, psychological capability, knowledge (perceived susceptibility, perceived severity).
			Social support (unspecified) (BCT 3.1)	Encourage social support(s) (e.g. partner, close family member, close friend) to provide advice, trigger or enable the decision-making process to initiate recommended health actions.
			Social support (emotional) (BCT 3.3)	
	OPPORTUNITY – Social			
	**Social influences**	**Enablement, Environmental restructuring, Modelling, Restriction**	Identification of self as role model (BCT 13.1)	Utilise women's desire to be a good role model for their children and other family members as a stimulus to trigger health action.
			Prompts/cues (BCT 7.1)	As per capability, psychological capability, knowledge (perceived susceptibility).
			Adding objects to the environment (BCT 12.5)	As per opportunity, physical opportunity, environmental context and resources (cues to action).

			Social support (unspecified) (BCT 3.1)	As per opportunity, physical opportunity, environmental context and resources (cues to action).
			Social support (emotional) (BCT 3.3)	
	MOTIVATION – Reflective			
	**Social/professional role**	**Education, Modelling, Persuasion**	Information about health consequences (BCT 5.1)	As per capability, psychological capability, knowledge (perceived susceptibility).
			Credible source (BCT 9.1)	As per capability, psychological capability, knowledge (perceived susceptibility).
			Prompts/cues (BCT 7.1)	As per capability, psychological capability, knowledge (perceived susceptibility).
			Identification of self as role model (13.1)	As per opportunity, social opportunity, social influences (cues to action).
	**Goals**	**Education, Coercion, Enablement, Modelling, Persuasion, Incentivisation**	Adding objects to the environment (BCT 12.5)	As per opportunity, physical opportunity, environmental context and resources (cues to action).
			Information about health consequences (BCT 5.1)	As per capability, psychological capability, knowledge (perceived susceptibility).
			Credible source (BCT 9.1)	As per capability, psychological capability, knowledge (perceived susceptibility).
			Information about social and environmental consequences (BCT 5.3)	As per capability, psychological capability, knowledge (perceived susceptibility).
			Prompts/cues (BCT 7.1)	As per capability, psychological capability, knowledge (perceived susceptibility).
			Social support (unspecified) (BCT 3.1)	As per opportunity, physical opportunity, environmental context and resources (cues to action).
			Social support (emotional) (BCT 3.3)	

	**Intentions**	**Education, Coercion, Incentivisation, Modelling, Persuasion**	Information about health consequences (BCT 5.1)	As per capability, psychological capability, knowledge (perceived susceptibility).
			Credible source (BCT 9.1)	As per capability, psychological capability, knowledge (perceived susceptibility).
			Information about social and environmental consequences (BCT 5.3)	As per capability, psychological capability, knowledge (perceived susceptibility).
			Prompts/cues (BCT 7.1)	As per capability, psychological capability, knowledge (perceived susceptibility).
	**Beliefs about capabilities**	**Education, Enablement, Modelling, Persuasion**	Adding objects to the environment (BCT 12.5)	As per opportunity, physical opportunity, environmental context and resources (cues to action).
			Information about health consequences (BCT 5.1)	As per capability, psychological capability, knowledge (perceived susceptibility).
			Credible source (BCT 9.1)	As per capability, psychological capability, knowledge (perceived susceptibility).
			Information about social and environmental consequences (BCT 5.3)	As per capability, psychological capability, knowledge (perceived susceptibility).
			Prompts/cues (BCT 7.1)	As per capability, psychological capability, knowledge (perceived susceptibility).
			Social support (unspecified) (BCT 3.1)	As per opportunity, physical opportunity, environmental context and resources (cues to action).
			Social support (emotional) (BCT 3.3)	
	**Beliefs about consequences**	**Education, Persuasion, Modelling**	Information about health consequences (BCT 5.1)	As per capability, psychological capability, knowledge (perceived susceptibility).
			Credible source (BCT 9.1)	As per capability, psychological capability, knowledge (perceived susceptibility).

			Information about social and environmental consequences (BCT 5.3)	As per capability, psychological capability, knowledge (perceived susceptibility).
			Prompts/cues (BCT 7.1)	As per capability, psychological capability, knowledge (perceived susceptibility).
	MOTIVATION – Automatic			
	**Emotion**	**Coercion, Enablement Incentivisation, Modelling, Persuasion**	Adding objects to the environment (BCT 12.5)	As per opportunity, physical opportunity, environmental context and resources (cues to action).
			Information about health consequences (BCT 5.1)	As per capability, psychological capability, knowledge (perceived susceptibility).
			Credible source (BCT 9.1)	As per capability, psychological capability, knowledge (perceived susceptibility).
			Information about social and environmental consequences (BCT 5.3)	As per capability, psychological capability, knowledge (perceived susceptibility).
			Social support (unspecified) (BCT 3.1)	As per opportunity, physical opportunity, environmental context and resources (cues to action).
			Social support (emotional) (BCT 3.3)	
	**Reinforcement**	**Training, Incentivisation, Coercion, Environmental restructuring**	Adding objects to the environment (BCT 12.5)	As per opportunity, physical opportunity, environmental context and resources (cues to action).
			Prompts/cues (BCT 7.1)	As per capability, psychological capability, knowledge (perceived susceptibility).
			Social support (unspecified) (BCT 3.1)	As per opportunity, physical opportunity, environmental context and resources (cues to action).
			Social support (emotional) (BCT 3.3)	
	CAPABILITY – Knowledge			

**Perceived benefits:**<br><br>• Improving personal health and health of baby.	**Knowledge**	**Education**	Information about health consequences<br>(BCT 5.1)	As per capability, psychological capability, knowledge (perceived barriers).<br><br>Additionally, explain how engaging in a healthy lifestyle (nutrition, physical activity, mental and emotional wellbeing) can also improve health outcomes for foetus and baby once born. To be spoken in an empowering and motivating way.
			Credible source<br>(BCT 9.1)	As per capability, psychological capability, knowledge (perceived susceptibility).
			Information about social and environmental consequences<br>(BCT 5.3)	As per capability, psychological capability, knowledge (perceived susceptibility).
			Prompts/cues<br>(BCT 7.1)	As per capability, psychological capability, knowledge (perceived susceptibility).
	**MOTIVATION – Reflexive**			
	**Beliefs about consequences**	**Education, Persuasion, Modelling**	Information about health consequences<br>(BCT 5.1)	As per capability, psychological capability, knowledge (perceived benefits).
			Credible source<br>(BCT 9.1)	
			Information about social and environmental consequences<br>(BCT 5.3)	As per capability, psychological capability, knowledge (perceived susceptibility).
			Identification of self as role model<br>(13.1)	Facilitate identification of self as role model. Inform women that engaging in a healthy lifestyle sets a good example for their children and broader family members, thereby helping reducing cardiometabolic disease risk in themselves, their children and broader family members.
			Feedback of outcome(s) of behaviour<br>(BCT 2.7)	As per capability, physical capability, physical skills (self-efficacy).
	**Beliefs about capabilities**	**Education, Enablement, Modelling, Persuasion**	Information about health consequences<br>(BCT 5.1)	As per capability, psychological capability, knowledge (perceived benefits).
			Credible source<br>(BCT 9.1)	As per capability, psychological capability, knowledge (perceived susceptibility).

			Feedback of outcome(s) of behaviour (BCT 2.7)	As per capability, physical capability, physical skills (self-efficacy).
			Information about social and environmental consequences (BCT 5.3)	As per capability, psychological capability, knowledge (perceived susceptibility).
			Self-monitoring of outcome(s) of behaviour (BCT 2.4)	Establish a method for women to monitor and record the outcomes of their health behaviours to reinforce benefits (e.g. hours restful sleep, mood, energy levels, weight, blood pressure). Encourage use of a journal to record and monitor outcomes overtime.
	**Optimism**	**Education, Enablement, Modelling, Persuasion**	Information about health consequences (BCT 5.1)	As per capability, psychological capability, knowledge (perceived benefits).
			Credible source (BCT 9.1)	As per capability, psychological capability, knowledge (perceived susceptibility).
			Feedback of outcome(s) of behaviour (BCT 2.7)	As per capability, physical capability, physical skills (self-efficacy).
			Information about social and environmental consequences (BCT 5.3)	As per capability, psychological capability, knowledge (perceived susceptibility).
			Self-monitoring of behaviour (BCT 2.3)	As per motivation, reflexive motivation, beliefs about capabilities (perceived benefits).
